# From cigarettes to symptoms: the association between smoking and depression in the German National Cohort (NAKO)

**DOI:** 10.1186/s12889-025-25959-0

**Published:** 2025-12-19

**Authors:** Maja P. Völker, Carolin M. Callies, Josef Frank, Jerome C. Foo, Iris Reinhard, Lea Zillich, Johanna Klinger-König, Hans Jörgen Grabe, Achim G. Beule, Angelika Erhardt-Lehmann, Alexander Pabst, Steffi G. Riedel-Heller, Bernhard T. Baune, Claudia Trenkwalder, Michael Wagner, Lilian Krist, Thomas Keil, Tobias Pischon, Katharina Nimptsch, Matthias B. Schulze, Börge Schmidt, Rafael Mikolajczyk, Nadia Obi, Volker Harth, Carolina J. Klett-Tammen, Heiko Becher, Karin H. Greiser, André Karch, Sabine Schipf, Claudia Meinke-Franze, Patricia Bohmann, Michael Leitzmann, Hermann Brenner, Ute Mons, Emanuel Schwarz, Klaus Berger, Jutta Mata, Stephanie H. Witt, Fabian Streit

**Affiliations:** 1 Department of Genetic Epidemiology in Psychiatry, Central Institute of Mental Health, Medical Faculty Mannheim, Heidelberg University, Mannheim, Germany; 2 Centre for Mental Health (DZPG), Partner Site Mannheim-Heidelberg-Ulm, Mannheim, Germany; 3 Health Psychology, School of Social Sciences, University of Mannheim, Mannheim, Germany; 4 Department of Psychiatry, College of Health Sciences, University of Alberta, Edmonton, AB Canada; 5 Neuroscience and Mental Health Institute, University of Alberta, Edmonton, AB Canada; 6 Core Facility Biostatistics, Central Institute of Mental Health, Medical Faculty Mannheim, Heidelberg University, Mannheim, Germany; 7 Department of Psychiatry and Psychotherapy, Medical Center – University of Freiburg, Faculty of Medicine, University of Freiburg, Freiburg, Germany; 8 Department of Psychiatry and Psychotherapy, University Medicine Greifswald, Greifswald, Germany; 9 Department of Otorhinolaryngology, Head and Neck Surgery, University Medicine Münster, Münster, Germany; 10 Max Planck Institute for Psychiatry, Munich, Germany; 11 Institute of Social Medicine, Occupational Health and Public Health, Medical Faculty, University of Leipzig, Leipzig, Germany; 12 Department of Psychiatry, University of Münster, Münster, Germany; 13 Paracelsus -Elena Hospital, Kassel, Germany; 14 Dept. Neurosurgery, University Medical Center Goettingen, Goettingen, Germany; 15 German Center for Neurodegenerative Diseases (DZNE), Bonn, Germany; 16 Department of Neurodegenerative Disease and Geriatric Psychiatry, University of Bonn Medical Center, Bonn, 53127 Germany; 17 Institute of Social Medicine, Epidemiology and Health Economics, Charité - Universitätsmeditzin Berlin, Berlin, Germany; 18 Institute of Clinical Epidemiology and Biometry, University of Würzburg, Würzburg, Germany; 19 State Institute of Health I, Bavarian Health and Food Safety Authority, Erlangen, Germany; 20 Max Delbrück Center for Molecular Medicine in the Helmholtz Association (MDC), Molecular Epidemiology Research Group, Berlin, Germany; 21 Max Delbrück Center for Molecular Medicine in the Helmholtz Association (MDC), Biobank Technology Platform, Berlin, Germany; 22 Charité - Universitätsmedizin Berlin, corporate member of Freie Universität Berlin and Humboldt-Universität zu Berlin, Berlin, Germany; 23 Department of Molecular Epidemiology, German Institute of Human Nutrition Potsdam-Rehbruecke, Nuthetal, Germany; 24 Institute of Nutritional Science, University of Potsdam, Nuthetal, Germany; 25 Institute for Medical Informatics, Biometry and Epidemiology, University of Duisburg Essen, University Hospital of Essen, Essen, Germany; 26 Institute for Medical Epidemiology, Biometrics, and Informatics, Interdisciplinary Center for Health Sciences, Medical Faculty of the Martin, Luther University Halle-Wittenberg, Halle (Saale), Germany; 27 German Center for Mental Health, Site Halle-Jena-Magdeburg, Halle (Saale), Germany; 28 Institute for Occupational and Maritime Medicine (ZfAM), University Medical Center Hamburg-Eppendorf, Hamburg, Germany; 29 Dept. of Epidemiology, Helmholtz Centre for Infection Research, Braunschweig, Germany; 30 Heidelberg Institute of Global Health, University Hospital Heidelberg, Heidelberg, Germany; 31 German Cancer Research Center (DKFZ) Heidelberg, Division of Cancer Epidemiology, Heidelberg, Germany; 32 Institute of Epidemiology and Social Medicine, University of Münster, Münster, Germany; 33 Institute for Community Medicine, University Medicine Greifswald, Greifswald, Germany; 34 Department of Epidemiology and Preventive Medicine, University of Regensburg, Regensburg, Germany; 35 German Cancer Research Center (DKFZ), Heidelberg, Germany; 36 Division Primary Cancer Prevention, German Cancer Research Center (DKFZ), Heidelberg, Germany; 37 Medical Faculty Mannheim, Heidelberg University, Mannheim, Germany; 38 Department of Psychiatry and Psychotherapy, Medical Faculty Mannheim, Central Institute of Mental Health, Heidelberg University, Mannheim, Germany; 39 Hector Institute for Artificial Intelligence in Psychiatry, Central Institute of Mental Health, Medical Faculty Mannheim, Heidelberg University, Mannheim, Germany

**Keywords:** Cigarette smoking, Depressive disorder, Smoking cessation, Age of onset

## Abstract

**Background:**

Although the association between smoking and depression is well-established, the underlying mechanisms and contextual factors remain insufficiently understood. We examined the association between smoking and depression, including detailed dose-response and timing-related relationships, using baseline data from a large population-based cohort, the German National Cohort (NAKO).

**Methods:**

The analysis comprised 173,890 participants (19-72 years, 50.21% female). Lifetime and current depression were assessed via self-reported physician’s diagnosis, the Major Depressive Disorder module of the MINI International Neuropsychiatric Interview (MINI), and the depression scale of the Patient Health Questionnaire (PHQ-9). Smoking behavior was assessed using self-reported smoking status, age at initiation, cigarettes per day, and time since smoking cessation. Associations between smoking and depression measures were analyzed using regression models adjusted for sex, age, age², education, Body Mass Index, and alcohol consumption.

**Results:**

Lifetime depression was more prevalent among individuals who currently or formerly smoked compared to those who never smoked. Currently smoking individuals also reported most current depressive symptoms, followed by formerly smoking individuals and those who never smoked. A dose-response relationship was observed, with more cigarettes per day being associated with more current depressive symptoms. Later age at smoking initiation was associated with later depression onset. Time since smoking cessation was positively associated with time since last depressive episode and negatively with current depressive symptoms.

**Conclusions:**

Our findings support an association between smoking and depression. Robust dose-response relationships were found, with higher cigarette consumption associated with more severe depressive symptoms, and longer time since cessation linked to lower depression levels. These results highlight smoking as a meaningful and modifiable contributor to current and lifetime depression, suggesting that quitting smoking or reducing cigarette consumption may benefit mental health. Early prevention of smoking initiation, along with integrated approaches that combine smoking cessation support with mental health care, may help reduce both smoking rates and depression burden.

**Supplementary Information:**

The online version contains supplementary material available at 10.1186/s12889-025-25959-0.

## Introduction

Smoking is the leading preventable cause of premature mortality worldwide [1]. Early initiation is associated with poorer health outcomes [2, 3], higher risk for nicotine dependence [4], and greater difficulties quitting [5]. Additionally, smoking is increasingly recognized for its impact on mental health, particularly depression [6–8]. Potential mechanisms include altered neurochemical pathways [9], systemic inflammation [10], and disrupted stress regulation [11]. These findings underline the importance of examining the implications of smoking for depression.

Major depressive disorder (MDD) is characterized by depressed mood and loss of interest or pleasure in activities ( [12]. Depression impairs daily functioning and quality of life [13], and imposes a high societal burden [14, 15]. The relationship between smoking and depression is complex [8, 16]; with evidence suggesting that smoking may contribute to depressive symptoms [8, 16, 17], while depression may also precede smoking initiation [16] and impact smoking severity [18]. Cross-sectional studies suggest that individuals who smoke daily face the highest risk of depression compared to those who occasionally smoke [19]. Conversely, a longitudinal study found that reducing cigarette consumption decreases depressive symptoms [20]. These findings support a dose-response relationship, suggesting that heavier smoking is associated with more severe depressive symptoms. Additionally, earlier age at smoking initiation is linked to earlier depression onset [3]. Conversely, smoking cessation is associated with improved depression outcomes [21, 22], with a more than 40% reduction in depression risk after 30 years of smoking cessation compared to current smoking [6] and a 2% decrease in the likelihood of depression per year [19].

Despite growing evidence on the link between smoking and depression, important gaps remain. Most prior studies are cross-sectional or based on small to medium samples, limiting the ability to clarify whether smoking contributes to the onset or worsening of depression, or whether depression drives smoking behaviors [23]. Moreover, different dimensions of smoking such as severity, age at initiation, and time since cessation have rarely been examined together, leaving dose-response and timing effects insufficiently understood. Large population-based studies that can evaluate these associations across sociodemographic subgroups are needed to clarify these aspects, particularly regarding the trajectory over time of the association between smoking behaviors and cessation with depression outcomes [24].

**Fig. 1:**
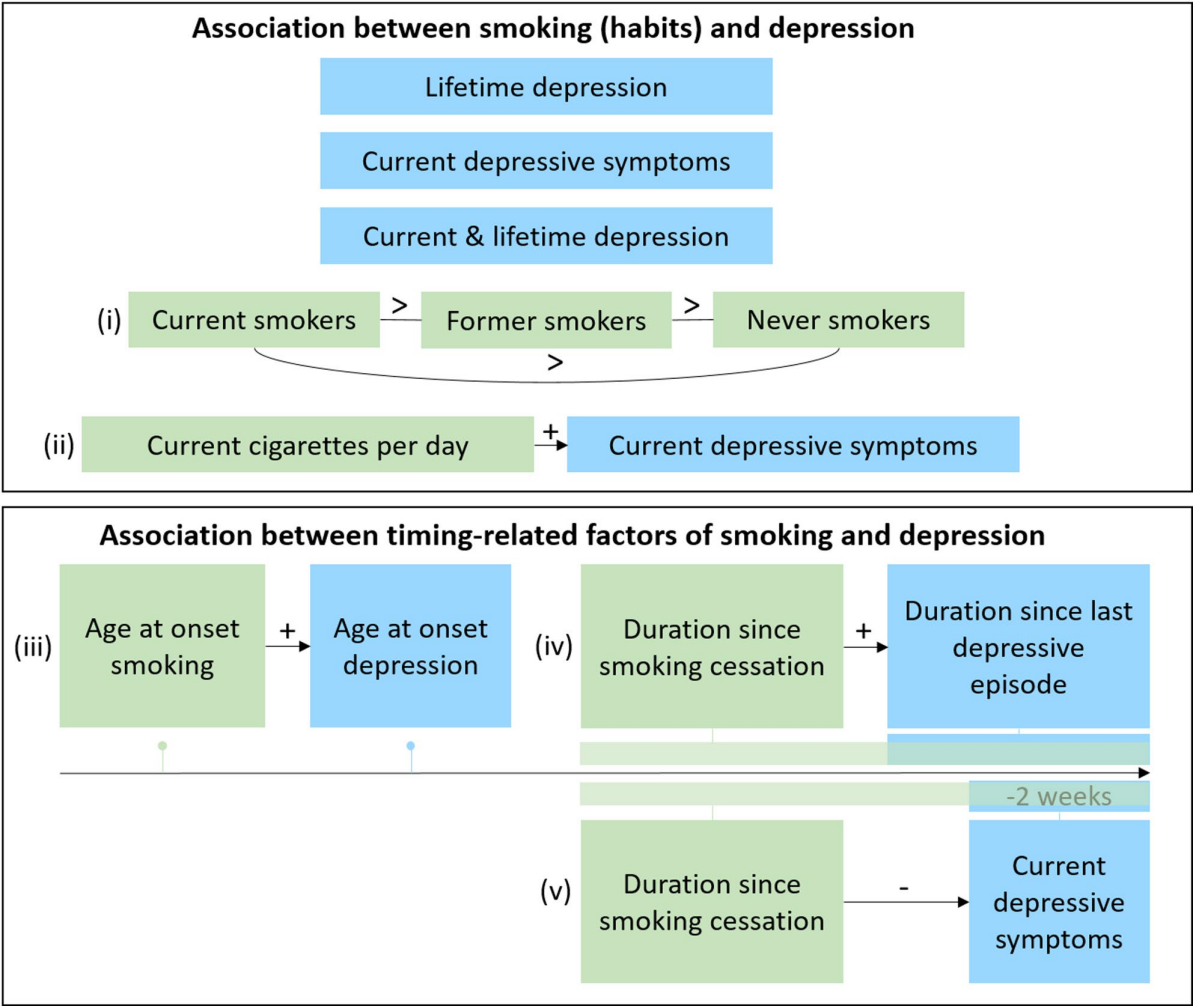
Visualization of hypothesized associations. Note. Green represents the predictor variable smoking. Blue represents the response variable depression. Arrow = direction. + = positive association. - = negative association. > = greater than

To address these gaps, we examined associations between smoking status, severity, onset, and cessation with depression outcomes using baseline data of the German National Cohort (NAKO), comprising data from more than 200,000 participants [25]. Given that the majority of currently and formerly smoking individuals in the NAKO reported smoking initiation prior to depression onset (93.8%), the present study focuses on the effects of smoking on depression outcomes. Incorporating timing and dose-response effects, we hypothesized that: (i) smoking is associated with an increased likelihood of current and lifetime depression, (ii) a higher number of cigarettes per day is associated with more severe current depressive symptoms, (iii) an earlier age at initiation of smoking is associated with an earlier age at onset of depression, (iv) a longer time since smoking cessation is associated with a longer time since the last depressive episode, and (v) a longer time since smoking cessation is associated with reduced severity of current depressive symptoms (Fig. 1). Additionally, analyses were repeated stratified by sex, age group, and education level to examine whether the observed associations were consistent across subgroups in accordance with well-established sociodemographic differences in both smoking behavior and depression.

## Methods

### Sample

The NAKO is a population-based longitudinal German cohort study investigating causes and trajectories of common diseases. Between 2014 and 2019, baseline data were collected for 205,415 participants aged 19 to 74 across 18 study centers. Inclusion criteria encompassed the ability to provide informed consent, sufficient knowledge of the German language, and the completion of a minimum set of assessments and questionnaires. Participants were selected randomly from local registries stratified by age and sex, with a higher proportion of participants aged 40 and above since the incidence of many investigated diseases within the NAKO peaks beyond the age of 40 [26]. All participants underwent a Level-1 (L1) assessment, including physical and cognitive examinations, a structured interview, touchscreen questionnaires, and biomaterial collection. A subset of approximately 28% underwent a more comprehensive Level-2 (L2) assessment including more physical examinations and instruments, among others the Mini International Neuropsychiatric Interview analyzed in the present study (see Lifetime depression) [25]. All local ethical committees provided ethical approval, and participants provided written informed consent in accordance with the Declaration of Helsinki.

### Measures

#### Cigarette smoking

Cigarette smoking was self-reported using a touchscreen questionnaire. First, participants were asked whether they had ever smoked (at least 100 cigarettes during their lifetime). Subsequently, they were categorized as having never smoked, having formerly smoked, or currently smoking. For currently and formerly smoking individuals, the age of smoking initiation was calculated from the age or year of smoking initiation. Additionally, they indicated the average current and former number of cigarettes per day. For formerly smoking individuals, time since smoking cessation in years was calculated from the age or year of quitting smoking. Other tobacco products (i.e., cigars, cigarillos, pipes) were excluded from the analyses.

#### Lifetime depression

For lifetime depression, participants were asked if they had ever received a depression diagnosis from a physician (physician’s diagnosis). If applicable, participants were asked to specify the year of or age at the diagnosis to calculate the age at onset of depression.

Additionally, the Major Depression module of the Mini International Neuropsychiatric Interview Version 5 (MINI) was used [27]. An initial filter question assessed the occurrence of periods of two weeks or more of feeling depressed or disinterested across the lifespan. Affirmative responses led to further questions about the last occurrence of a lifetime depressive episode (month and year). For L2 participants, the presence of at least one cardinal symptom triggered the remaining questions for symptomatology and impairment level. If five or more symptoms applied, a positive MINI classification was assigned; otherwise a negative MINI classification was assigned. A more detailed description can be found elsewhere [28].

#### Current depressive symptoms

For current depressive symptoms, participants completed the depression module of the Patient Health Questionnaire (PHQ-9) with nine items about the presence and frequency of depression symptoms in the past two weeks according to the DSM-IV. Responses ranged from 0 (‘not at all’) to 3 (‘almost every day’). The sum of all items indicated the severity of current depressive symptoms (0 to 27). Additionally, a cut-off score of ≥ 10 (PHQ-9 ≥ 10) indicated the presence of a moderate to severe current depressive episode [29].

#### Current depressive symptoms and lifetime diagnosis

To account for the presence of both lifetime and current depression simultaneously, a variable was constructed with the physician’s diagnosis and the PHQ-9 cutoff with four levels: *no current symptoms & no lifetime diagnosis* (PHQ-9 sum score < 10 & no diagnosis), *lifetime diagnosis only* (PHQ-9 sum score < 10 & diagnosis), *current symptoms only* (PHQ-9 sum score ≥ 10 & no diagnosis), *current symptoms & lifetime diagnosis* (PHQ-9 sum score ≥ 10 & diagnosis).

#### Covariates

All models included sex, age and age² (continuous), education level, Body Mass Index (BMI), and alcohol consumption as covariates. For sex, biological sex was assessed. The age was derived from the date of birth subtracted from the day of the examination. Education was classified according to the International Standard Classification of Education 1997 (ISCED 97) [30, 31] into lower (1–2), medium (3–4), and higher (5–6). An additional category, ‘in progress,’ was added since many (mostly younger) participants indicated still being in training. The BMI was calculated from the height and weight measured at the day of the examination. Alcohol consumption was assessed using the short version of the Alcohol Use Disorders Identification Test (AUDIT-C) [32]. The AUDIT-C comprises three questions regarding the frequency of alcohol consumption, alcohol quantity on a typical drinking day and the frequency of binge drinking. Items are rated on a 5-point Likert scale ranging from 0 to 4. For the analyses, the AUDIT-C was scored continuously ranging from 0 to 12 points.

### Statistical analysis

**Fig. 2:**
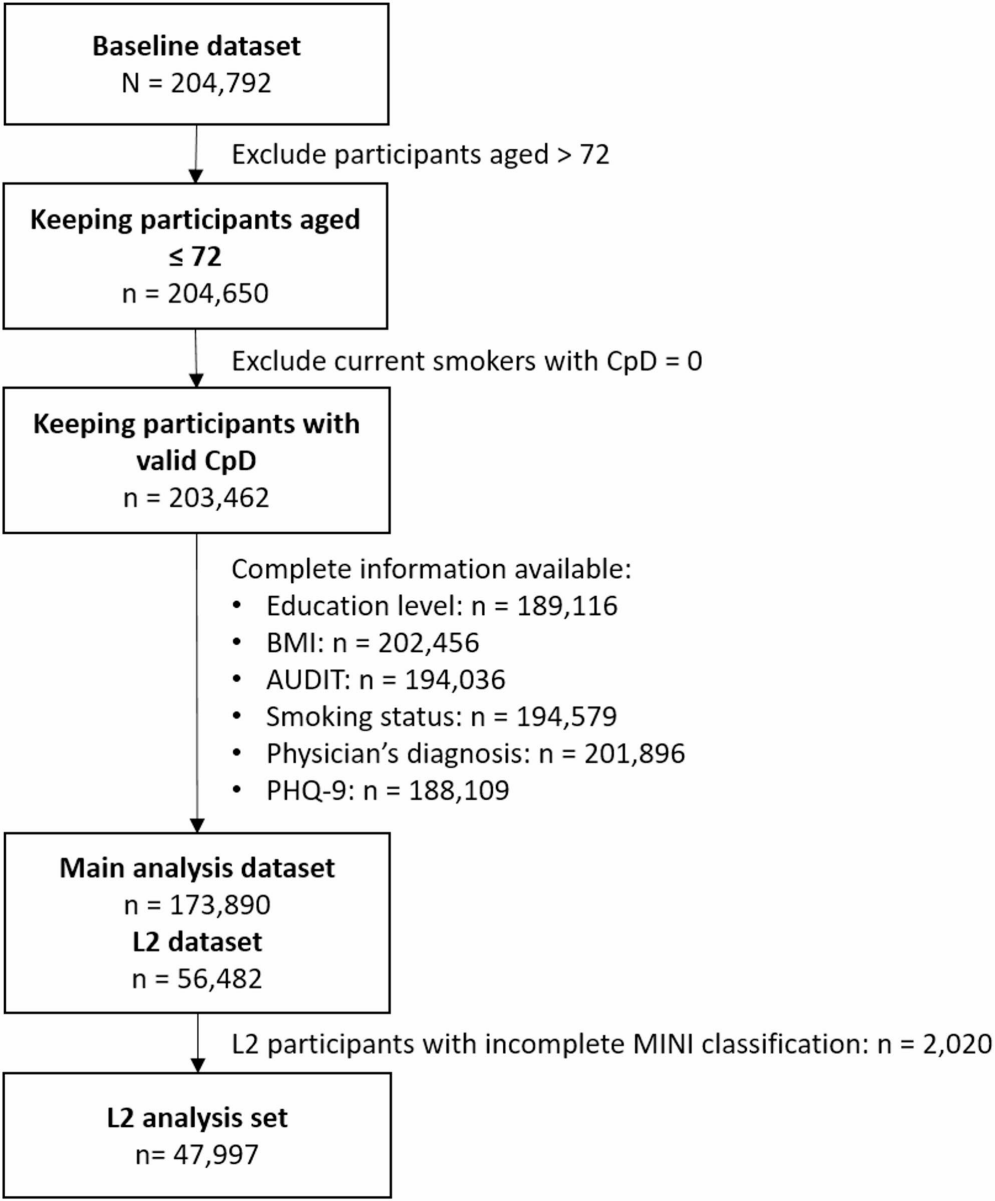
Exclusion flow chart. Note. CpD = Cigarettes per day. BMI = Body Mass Index. AUDIT = Alcohol Use Disorders Identification Test. PHQ-9 = Patient Health Questionnaire. L2 = Level-2. MINI = Mini International Neuropsychiatric Interview. MINI classification was available for L2 participants only. Participants with missing values on any relevant variable were excluded by listwise deletion

The present analyses were performed with R (v4.2.1). All analyses were exploratory. Using listwise deletion, participants older than 72 years or with missing values on any variable of interest (smoking variables, physician’s diagnosis, PHQ-9, covariates) were excluded from the analyses, resulting in a final analysis sample of 173,890 (see Fig. 2). For testing the association between age at smoking initiation and depression onset, analyses were restricted to formerly and currently smoking individuals who initiated smoking before the first depressive episode. For testing the association between the time since smoking cessation and since last depressive episode, analyses were restricted to formerly smoking individuals who quit smoking before their last depressive episode. Linear and logistic regression models were applied to test associations of smoking measures with continuous and categorical depression outcomes, respectively (see Table 1 and Supplement text S1). For linear models, estimated marginal means (EMM), and for logistic models, Odds Ratios (OR), and adjusted frequencies (estimated from the respective regression model) were calculated, including their respective 95% confidence intervals (CI). All regression models were adjusted by sex, age, age² (to account for non-linear age effects in depression [e.g. 28, 33]), education level, Body Mass Index (BMI), and alcohol consumption. An overview of all tested associations is shown in Fig. 1 and Table 1. Bonferroni correction was applied with a significance threshold of *p* ≤ .006 (α = 0.05/8 tests). As most tested associations were highly significant (*p* < 10^− 10^), precise p-values are reported only for those with *p* > 10^− 10^. Analyses were repeated, stratified by sex; by age group (19–29, 30–39, 40–49, 50–59, 60–72 years), assuming stronger effects in older age groups due to longer smoking exposure; and by education level (in progress, low, medium, high), given that low socioeconomic status (SES) is associated with both smoking [34] and depression [35].

**Table 1:** Overview of all statistical analyses with predictors and outcomes

	Subgroup	Regression analysis	Predictor	Outcome
(i)	Current, former & never	Multiple binary logistic	Smoking status *[categorical: ‘never’*,* ‘former’*,* ‘current’]*	a) Physician’s diagnosis of depression *[categorical: ‘yes’*,* ‘no’]* b) MINI classification *[categorical: ‘positive’*,* ‘negative’]*
(i)	Current, former & never	Multiple binary logistic	Smoking status *[categorical: ‘never’*,* ‘former’*,* ‘current’]*	Current depressive symptoms *[categorical: ‘PHQ-9 ≥ 10’*,* ‘PHQ-9 < 10’]*
(i)	Current, former & never	Multinomial logistic	Smoking status *[categorical: ‘never’*,* ‘former’*,* ‘current’]*	Physician’s diagnosis & current depressive symptoms *[categorical: ‘PHQ-9 < 10 & no diagnosis’ ´PHQ-9 ≥ 10 & no diagnosis´*,* ´ PHQ-9 < 10 & diagnosis´*,* ´PHQ-9 ≥ 10 & diagnosis´]*
(ii)	Current	Multiple linear	Cigarettes per day *[continuous]*	Current depressive symptoms *[continuous: PHQ-9 sum score]*
(iii)	Current & former	Multiple linear	AAO of smoking *[continuous]*	AAO of depression *[continuous: AAO of physician’s diagnosis]*
(iv)	Former	Multiple linear	Time since smoking cessation *[continuous]*	Time since last depressive episode *[continuous: time reported in MINI Screen]*
(v)	Former	Multiple linear	Time since smoking cessation *[continuous]*	Current depressive symptoms *[continuous: PHQ-9 sum score]*

MINI = MINI International Neuropsychiatric Interview. PHQ-9 = Patient Health Questionnaire. AAO = Age at onset. Square brackets indicate the measurement scale and levels of the categorical variable. All analyses were performed with sex, age, age², education, BMI, and alcohol consumption as covariates

## Results

### Descriptives

The initial data set included 204,792 participants. After listwise deletion of participants with missing values (see Fig. 2), 173,890 participants remained (84.9% of the total sample, 50.21% females, Øage = 49.21). 81,775 participants indicated having never smoked, 58,004 formerly smoked, and 34,111 currently smoked. 55.0% reported having high education, 40.6% medium education, 2.1% low education and 2.4% education in progress. For a detailed sample description, see Table 2.

**Table 2:** Descriptive statistics of covariates, smoking, and depression for the total sample and by smoking status

	Total (*n* = 173,890)	Never smoked (*n* = 81,775)	Formerly smoked (*n* = 58,004)	Currently smoking (*n* = 34,111)
Age	49.2 (12.8)	47.9 (13.4)	52.4 (11.6)	46.9 (12.2)
Sex female	50.2%	54.6%	45.9%	47.1%
Education high	55.0%	60.6%	54.4%	42.5%
Education medium	40.6%	34.7%	42.8%	51.2%
Education low	2.1%	1.4%	2.0%	3.9%
Education in progress	2.4%	3.4%	0.9%	2.4%
BMI	26.6 (5.0)	26.1 (4.9)	27.3 (5.1)	26.3 (5.0)
AUDIT-C sum score	3.4 (2.1)	3.0 (1.9)	3.7 (2.1)	3.9 (2.4)
Cigarettes per day (*n* = 92,115)	12.8 (9.8)	-	13.4 (10.4)	11.8 (8.5)
AAO of smoking in years (*n* = 91,950)	18.1 (8.5)	-	18.1 (6.2)	18.1 (6.2)
Time since smoking cessation in years (*n* = 57,972)	17.0 (12.2)	-	17.0 (12.2)	-
Physician’s diagnosis of depression	14.2%	11.5%	15.5%	18.2%
AAO Physician’s diagnosis of depression in years (*n* = 24,288)	39.3 (12.5)	39.3 (12.5)	40.5 (12.6)	37.6 (12.1)
MINI classification L2 positive (*n* = 47,997)	15.0%	12.2%	16.3%	19.9%
MINI time since last depressive episode in years (*n* = 32,199)	5.6 (7.7)	5.7 (7.6)	6.3 (8.2)	4.7 (6.8)
PHQ-9 sum score	3.9 (3.7)	3.7 (3.5)	3.9 (3.7)	4.6 (4.3)
PHQ-9 cutoff ≥ 10	7.7%	6.3%	7.4%	11.8%

Standard deviations are indicated in parentheses. BMI = Body-Mass-Index. AUDIT-C = Alcohol Use Disorders Identification Test. cigarettes per day = Cigarettes per day. AAO = Age at onset. MINI = MINI International Neuropsychiatric Interview. PHQ-9 = Patient Health Questionnaire

For the MINI classification, 47,997 L2 participants remained. For details, see Fig. 2 and Table 2.

### Association of smoking status with lifetime depression

**Fig. 3:**
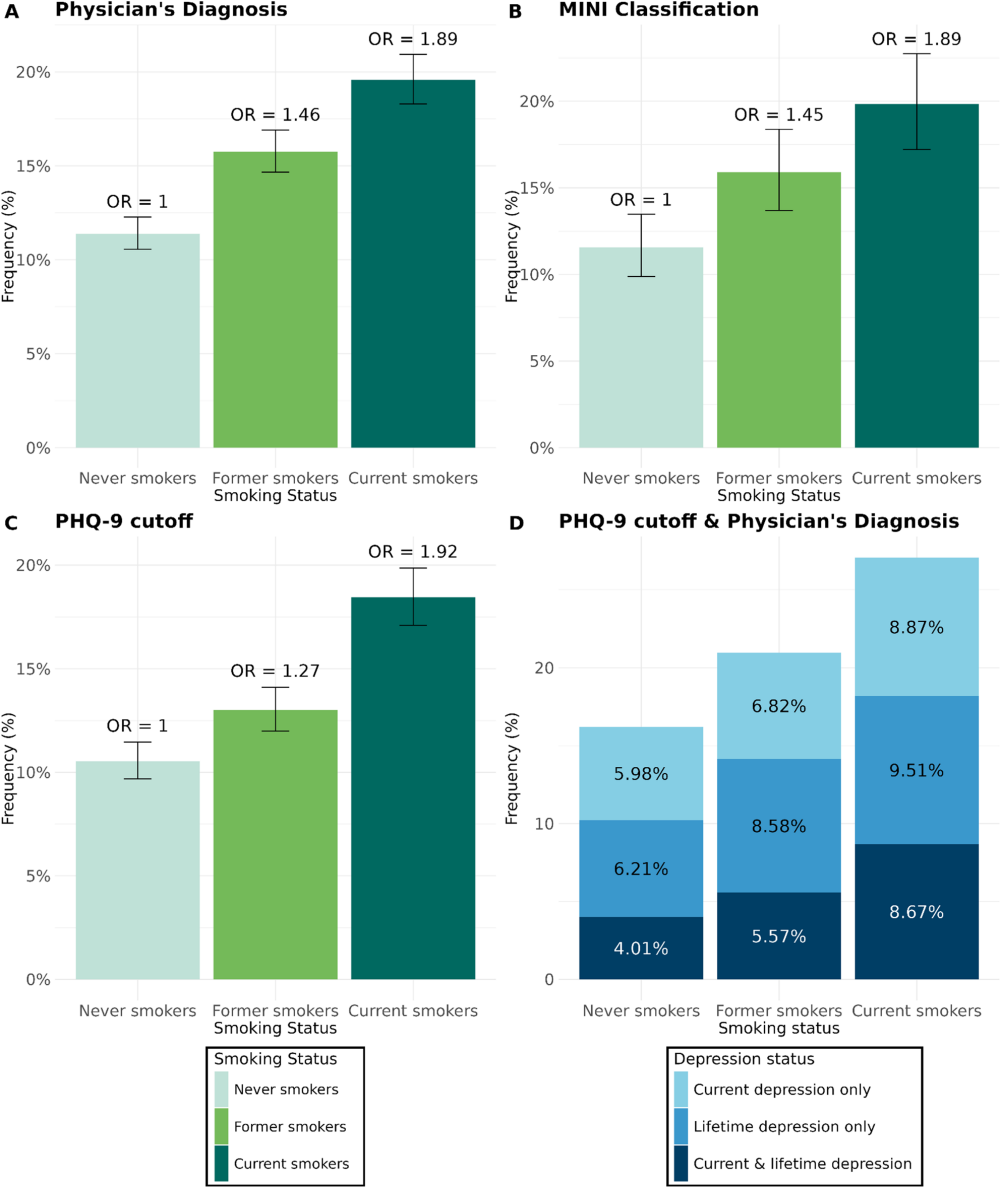
Adjusted Frequencies for Lifetime and Current Depression Measures by Smoking Status. **A**) Physician's diagnosis. **B**) MINI classification. **C**) PHQ-9 cutoff. **D**) PHQ-9 cutoff & physician's diagnosis. Note. OR = Odds ratio. PHQ-9 = Patient Health Questionnaire. Frequencies are adjusted for the covariates sex, age, age², education, BMI, and alcohol consumption. OR are reported in Panels **A**, **B**, and **C** with individuals who never smoked as the reference group

The adjusted frequency for physician’s diagnosis was 11.4% for individuals who never smoked (CI [10.6%,12.3%]), 15.8% for those who formerly smoked (CI [14.7%,16.9%]), and 19.6% for those who currently smoked (CI [18.3%, 20.9%]). Higher OR for currently (OR = 1.89, CI [1.83,1.97], *p* < 10^− 10^) followed by formely smoking individuals (OR = 1.46, CI [1.41,1.50], *p* < 10^− 10^) compared to individuals who never smoked were observed (Table S1; Fig. 3A). For the L2 participants, the adjusted frequency for a positive MINI classification for individuals who never smoked was 11.6% (CI [9.9%,13.5%]), 15.9% for those who formerly smoked (CI [14.0%,18.4%]), and 19.8% for those who currently smoked (CI [17.2%,22.8%]). Higher OR for currently (OR = 1.89, CI [1.77,2.02], *p <* 10^− 10^) and formerly smoking individuals (OR = 1.45, CI [1.36,1.54], *p <* 10^− 10^) compared to individuals who never smoked were observed (Table S2, Supplement text S2, Fig. 3B).

### Association of smoking status with current depression

The adjusted frequencies of a PHQ-9 cut-off ≥ 10 were 10.5% for individuals who never smoked (CI [9.7%,11.5%]), 13.0% for those who formerly smoked (CI [12.0%,14.1%]), and 18.4% (CI [17.1%,19.9%]) for those who currently smoked, corresponding to an OR of 1.92 (CI [1.83,2.01], *p <* 10^− 10^) in currently smoking individuals, and an OR of 1.27 (CI [1.22,1.33], *p <* 10^− 10^) in individuals who formerly smoked compared to those who never smoked (Fig. 3C, Table S3A, Supplement text S2). Additionally, smoking status was associated with PHQ-9 sum score (F(10, 173879) = 905.2, *p <* 10^− 10^): individuals who formerly smoked showed the highest mean PHQ-9 sum scores (*EMM* = 5.13, *SE* = 0.03), followed by individuals who formerly smoked (*EMM* = 4.55, *SE* = 0.03), and lastly individuals who never smoked (*EMM* = 4.22, *SE* = 0.02; Figure S1, Table S3B).

### Association of smoking status with lifetime and current depression

The adjusted frequencies for *current symptoms only* were 6.0% for individuals who never smoked (CI [5.9%,6.1%]), 6.8% for individuals who formerly smoked (CI [6.7%,6.9%]), and 8.9% for those who currently smoked (CI [8.7%,9.0%]). The adjusted frequencies for *lifetime diagnosis only* were 6.2% for individuals who never smoked (CI [6.1%,6.3%]), 8.6% for those who formerly smoked (CI [8.5%,8.7%]), and 9.5% for those who currently smoked (CI [9.4%,9.7%]). The adjusted frequencies for *current symptoms and lifetime diagnosis* were 4.0% for individuals who never smoked (CI [3.9%,4.1%]), 5.6% for those who formerly smoked (CI [5.5%,5.7%]), and 8.7% for those who currently smoked (CI [8.5%,8.8%]) (Fig. 3, Table S4, Supplement text S2).

### Association of smoking intensity and severity of current depressive symptoms

**Fig. 4:**
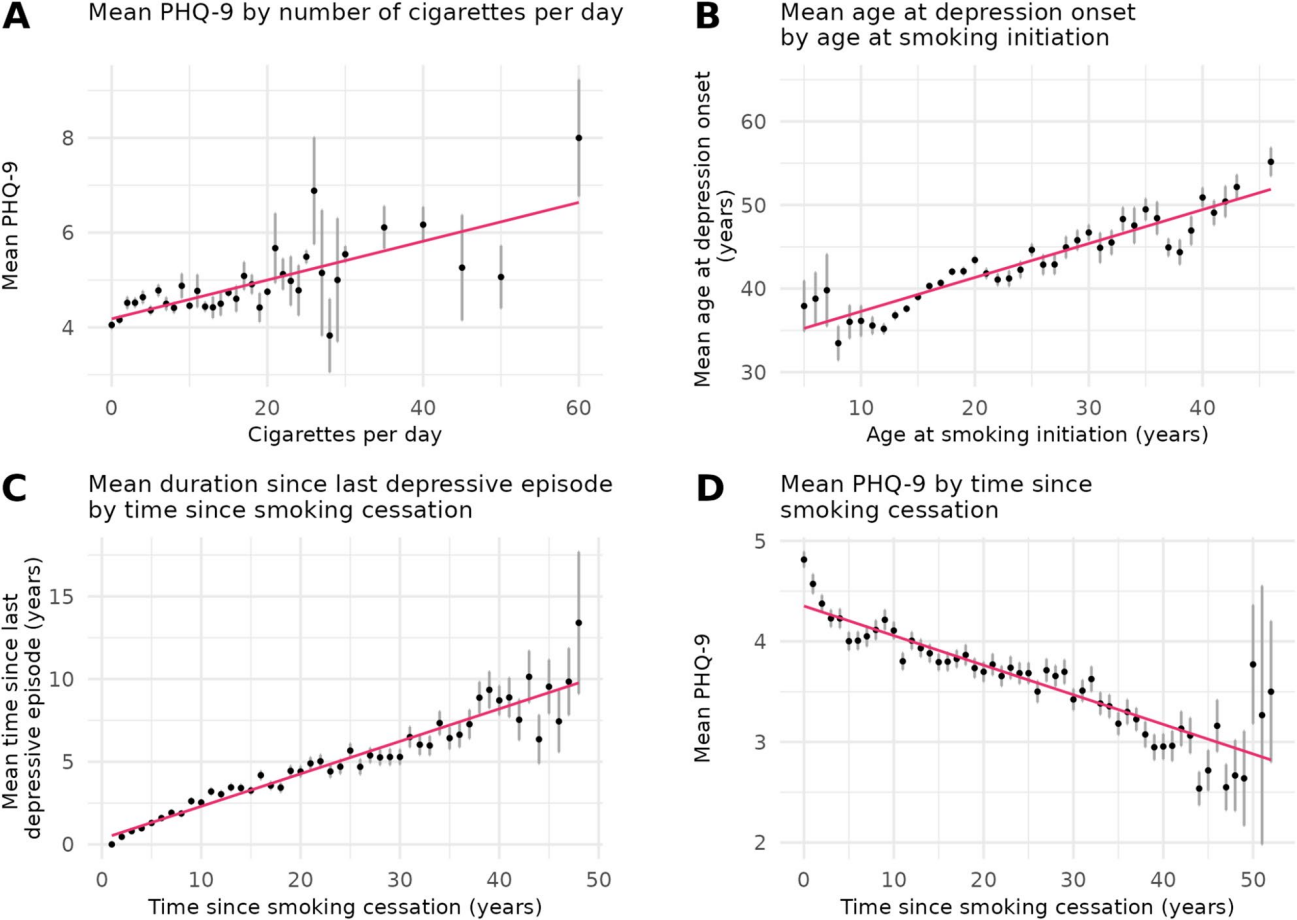
Descriptive means of smoking behaviors and temporal factors with depression outcomes. **A**) Mean PHQ-9 by number of cigarettes per day. **B**) Mean age at depression onset by age at smoking initiation. **C**) Mean duration since last depressive episode by time since smoking cessation. **D**) Mean PHQ-9 by time since smoking cessation. Note. PHQ-9 = Patient Health Questionnaire. Associations between mean levels of predictor and outcome are displayed. The black dots represent the descriptive mean of each outcome variable for each level of the respective predictor with 95% CIs. The unadjusted regression lines are displayed in pink

Among individuals who formerly smoked, the mean cigarettes per day was 11.8 (SD = 8.5) while the mean PHQ-9 sum score was 4.6 (SD = 4.3). A significant positive association was found, with a higher number of cigarettes per day associated with higher PHQ-9 sum scores (Fig. 4). The regression model explained 4.7% of variance in PHQ-9 sum score with cigarettes per day being a significant predictor (β = 0.05 symptoms per additional cigarette, CI [0.04,0.05], *p <* 10^− 10^, Table S5).

### Association of age at smoking initiation and depression onset

The mean age at smoking initiation was 18.1 years (SD = 6.2), while the mean age at depression onset was 39.3 years. Of 14,991 participants reporting both an age at smoking initiation and at depression onset, 14,060 participants indicated smoking initiation prior to depression onset (93.8%), 695 depression onset prior to smoking initiation (4.6%), and 236 reported the same age for both (1.6%). For those with smoking initiation before depression onset, a positive association between the ages at smoking initiation and depression onset was observed, with a younger age at smoking initiation being associated with a younger age at depression onset (Fig. 4). The model (containing the predictor of interest and covariates) explained 45.3% of variance in the age at depression onset, with age at smoking initiation being a significant predictor (β = 0.24, CI [0.21,0.27], *p <* 10^− 10^, Table S6A). Effect estimates were equal for individuals who formerly (β = 0.24, CI [0.20,0.28], *p <* 10^− 10^, Table S6B) and currently smoked (β = 0.24, CI [0.19,0.28], *p <* 10^− 10^, Table S6C).

### Association of time since smoking cessation and since the last depressive episode

The mean time since smoking cessation was 17.0 years (SD = 12.2), while the mean time since the last depressive episode was 6.3 years (SD = 8.2). A significant positive association was found, with a longer time since smoking cessation being associated with a longer time since the last depressive episode (Fig. 4). The regression model (containing the predictor of interest and covariates) explained 14.5% of variance in the time since the last depressive episode, with time since smoking cessation being a significant predictor (β = 0.17 years since last episode per year since cessation, CI [0.16,0.18], *p <* 10^− 10^, Table S7).

### Association of time since smoking cessation and severity of current depressive symptoms

For individuals who formerly smoked, the mean PHQ-9 sum score was 3.9 (SD = 3.7). A negative association between smoking cessation and PHQ-9 sum scores was observed, with a longer time since cessation being associated with lower PHQ-9 sum scores (Fig. 4). The regression model (containing the predictor of interest and covariates) explained 4.9% of the variance in PHQ-9 sum score with time since cessation being a significant predictor (β = -0.02 symptoms per year since cessation, CI [-0.02,-0.02], *p* < 10^*− 10*^, Table S8).

### Stratified analyses by sex, age group, and education level

Subgroup analyses by sex, age group, and education level largely mirrored the overall findings. Across nearly all subgroups, individuals who formerly smoked showed the strongest associations with lifetime and current depression, followed by individuals who formerly smoked, and lastly individuals who never smoked.

Some variations were observed: The age group 19–29 showed smaller differences between currently and formerly smoking individuals. In contrast, age groups 40–59 exhibited the clearest distinctions, with individuals who currently smoked consistently showing stronger associations than those who formerly smoked. Regarding education, individuals with education-in-progress showed a distinct pattern: individuals who formerly smoked had higher odds for lifetime depression than those who currently smoked. For analyses involving age at smoking initiation and depression onset, and time since smoking cessation and time since last depressive episode, effect estimates were slightly smaller or not significant in the education-in-progress group and the youngest age group. Full results for subgroup analyses are provided in the Supplementary Material (Supplement text S3-S8, Tables S9A–S15K, Figures S1–S22).

## Discussion

The present study examined the association between cigarette smoking and depression in a large cross-sectional data set with more than 170,000 people, including detailed analyses of dose-response relationships, and novel temporal associations between smoking and depression. The results show a robust association of smoking with the occurrence of depression and symptom severity: individuals who formerly smoked had the highest odds for lifetime and current depression, followed by those who formerly smoked, compared to those who never smoked; consistent across sexes, education levels, and age groups. A dose-response relationship was observed, with more cigarettes per day being associated with more severe current depressive symptoms. Importantly, earlier smoking initiation was associated with earlier depression onset, and a longer time since smoking cessation with a longer time since the last depressive episode, and less severe current depressive symptoms. Together, these findings extend prior work by demonstrating not only dose-dependent associations but also previously unexplored temporal patterns, pointing toward a temporally linked and dose-dependent relationship between smoking and depression.

The findings align with prior research indicating that currently and formerly smoking individuals are most likely to report a history of depression [6, 8, 36], with stronger effect estimates for lifetime depression in the present study (OR_current_ =1.89, CI [1.83,1.97]; OR_former_ =1.46, CI [1.41,1.50]) compared to a meta-analysis by Luger and colleagues (2014) (OR_current_ = 1.50, CI [1.29,1.60]; OR_former_ = 1.21, CI [1.13,1.30]). Differences might reflect heterogeneity in depression measures and characteristics (e.g., age distribution, sampling strategy) or lack of adjustment for age in some of the 78 studies in the meta-analysis. Participants with education in progress displayed a distinct pattern, where individuals who formerly smoked had a higher OR of lifetime depression than those who currently smoked. This could reflect underlying vulnerabilities, early cessation due to health problems, or statistical fluctuations due to the small group size.

Current depressive symptoms were higher in individuals who currently smoked followed by individuals who formerly smoked compared to those who never smoked. While research consistently showed higher depression rates in individuals who currently smoked compared to those who never smoked [6, 19, 37], findings regarding differences between formerly and never smoking individuals varied. Results might depend on the exact depression measures, investigated time frames, and study characteristics (e.g., age range, sampling method). Moreover, age distribution might have influenced the observed associations: In the age-stratified analyses, the differences between current and individuals who formerly smoked were less pronounced in the 19–29 age group, likely reflecting shorter cessation times, whereas larger differences emerged in the 40–49 and 50–59 age groups. Oversampling of individuals above 40 in the NAKO might have accentuated differences in the overall estimates.

Participants were additionally categorized into *no depression*, *current depressive symptoms only*, *lifetime diagnosis only*, and *current symptoms and lifetime diagnosis*. While individuals who never or formerly smoked showed similar frequencies of *current depressive symptoms only*, individuals who currently smoked showed higher frequencies, suggesting that current depressive symptoms are particularly linked to current smoking. Meanwhile, individuals who currently and formerly smoked show similar frequencies of *lifetime depression only* compared to individuals who never smoked, demonstrating that a history of smoking is associated with a history of depression during the lifetime. For *current and lifetime depression combined*, individuals who currently smoked showed the highest frequencies, followed by those who formerly smoked, compared to those who never smoked.

A potential negative effect of smoking on mental health was supported by an observed dose-response relationship with more cigarettes per day being associated with greater symptom severity. This aligns with research comparing individuals who smoke occasionally vs. daily [19], levels of nicotine dependence [38], the heaviness of smoking [39], or cumulative pack years [6], and biomarkers such as cotinine levels [40], suggesting a pharmacologic or neurobiological link between nicotine exposure and mood dysregulation. Therefore, even reductions in cigarette consumption (short of full cessation) might alleviate depressive symptoms.

The temporal analyses further indicated that earlier smoking initiation was associated with earlier depression onset, replicating prior research on early-onset smoking and depression risk [3, 41]. In the present study, the largest effect estimates were observed for the age group 19–29, potentially explained by stronger temporal coupling or more accurate self-reporting due to weaker recall bias in younger individuals.

Further, a longer time since smoking cessation was associated with a longer time since the last depressive episode, in line with prior studies suggesting that cessation is linked to improved depression outcomes [21, 42]: Hahad et al. [6] found a more than 40% reduction in depression risk after 30 years of cessation compared to individuals who currently smoked, and Wu et al. [21] identified a decrease of 2% in depression likelihood per additional year of cessation. Effect estimates were comparable across the stratified analyses except for the education-not-finished group, potentially confounded by the small subgroup size.

Additionally, a longer time since smoking cessation was associated with fewer depressive symptoms, in line with previous research demonstrating that a longer time since cessation was associated with a stronger decrease in depressive symptoms [19, 36, 43]. Stratified analyses were consistent across all age groups except for individuals aged 19–29, and across all education levels except for those with education in progress, coinciding with younger age groups. This suggests that the mental health benefits of quitting smoking may take longer to appear than many young people have been smoke-free, or that their shorter smoking history led to smaller mental health changes.

Strengths of this study include its large sample size, providing high statistical power for stratified analyses, and comprehensive measures capturing various aspects of smoking and depression. According to Bradford Hill’s criteria of temporality and biological gradient, the observed dose–response and timing patterns strengthen the plausibility of a causal relationship [44]; although reverse causality remains possible: depression may contribute to smoking initiation [8, 16] or hinder smoking cessation [45]. Longitudinal data or experimental validation is needed to confirm causality.

However, the cross-sectional study design and the retrospective assessments limit causal inference from the present analyses, and recall bias may have affected reports of smoking initiation, depression onset, and cessation history.

Although the NAKO spans multiple study sites across Germany and includes a broad age range, exact estimates may not be fully representative of the German population. For instance, despite a high response for a study of this scale (15.6%) [26], certain subgroups (e.g., lower SES or more severe mental health issues) may be underrepresented due to non-participation, although analyses were adjusted for education level as a proxy for SES, and the results of the stratified analyses were largely consistent across education levels. Similarly, the aforementioned subgroups might have been less likely to complete all assessments, and since participants with missings on the relevant variable were excluded from the analyses, this might have reinforced underrepresentation. Moreover, nicotine exposure from non-cigarette products was not examined here, and the directionality between smoking and depression cannot be fully disentangled given evidence that depression may influence smoking behavior [16].

Potential mechanisms linking smoking and depression may include a shared genetic predisposition [46] or alterations in brain regions involved in both conditions [9, 47]. Future longitudinal analyses, and integration of available fMRI and genetic data in the NAKO may help clarify these pathways [25, 48]. Additionally, the temporal association between smoking and depression could be strengthened by including a negative-control analysis, testing whether another behaviour commonly initiated at a similar age (e.g., alcohol use or social media use) also predicts later depression.

Overall, the present results revealed differences in lifetime and current depression frequencies according to smoking status. Beyond confirming an association between smoking and depression, our study demonstrates dose-dependent effects of smoking heaviness on depression severity, while longer time since cessation predicted both reduced symptoms and longer periods of remission. The results highlight the importance of preventing smoking initiation and promoting cessation, or harm-reduction strategies to support mental health. From a policy perspective, integrating mental health considerations into tobacco-control initiatives (e.g., cessation interventions in primary care) may enhance the effectiveness of public health approaches. Future research should further clarify the temporal and causal mechanisms underlying the smoking–depression link, and evaluate whether early intervention or harm-reduction strategies can mitigate mental health risks. Taken together, the present findings suggest that heavier smoking confers greater depressive burden, while sustained cessation yields mental health benefits, underscoring the need for early prevention and targeted cessation support.

## Supplementary Information

Supplementary Material 1.

Supplementary Material 2.

Supplementary Material 3.

## Data Availability

The data used for the analyses is available upon request at https://transfer.nako.de/.

## References

[1] World Health Organization. WHO Report on the Global Tobacco Epidemic. 2021. In: Security Research Hub Reports. digitalcommons.fiu.edu; 2021.

[2] Fa-Binefa M, Clará A, Pérez-Fernández S, Grau M, Dégano IR, Marti-Lluch R, et al. Early smoking-onset age and risk of cardiovascular disease and mortality. Prev Med. 2019;124:17–22. 10.1016/j.ypmed.2019.04.022. 31054906 10.1016/j.ypmed.2019.04.022

[3] Jamal M, der Does AJWV, Penninx BWJH, Cuijpers P. Age at smoking onset and the onset of depression and anxiety disorders. Nicotine Tob Res. 2011;13:809–19. 10.1093/ntr/ntr077. 21543549 10.1093/ntr/ntr077

[4] Kendler KS, Myers J, Damaj MI, Chen X. Early smoking onset and risk for subsequent nicotine dependence: a monozygotic co-twin control study. Am J Psychiatry. 2013;170:408–13. 10.1176/appi.ajp.2012.12030321. 23318372 10.1176/appi.ajp.2012.12030321 PMC3615117

[5] Khuder SA, Dayal HH, Mutgi AB. Age at smoking onset and its effect on smoking cessation. Addict Behav. 1999;24:673–7. 10.1016/s0306-4603(98)00113-0. 10574304 10.1016/s0306-4603(98)00113-0

[6] Hahad O, Beutel M, Gilan DA, Michal M, Schulz A, Pfeiffer N, et al. The association of smoking and smoking cessation with prevalent and incident symptoms of depression, anxiety, and sleep disturbance in the general population. J Affect Disord. 2022;313:100–9. 10.1016/j.jad.2022.06.083. 35777492 10.1016/j.jad.2022.06.083

[7] Yuan S, Yao H, Larsson SC. Associations of cigarette smoking with psychiatric disorders: evidence from a two-sample Mendelian randomization study. Sci Rep. 2020;10:13807. 10.1038/s41598-020-70458-4. 32796876 10.1038/s41598-020-70458-4 PMC7427799

[8] Luger TM, Suls J, Vander Weg MW. How robust is the association between smoking and depression in adults? A meta-analysis using linear mixed-effects models. Addict Behav. 2014;39:1418–29. 10.1016/j.addbeh.2014.05.011. 24935795 10.1016/j.addbeh.2014.05.011

[9] Hajdusianek W, Żórawik A, Waliszewska-Prosół M, Poręba R, Gać P. Tobacco and nervous system development and function-new findings 2015–2020. Brain Sci. 2021;11:797. 10.3390/brainsci11060797. 34208753 10.3390/brainsci11060797 PMC8234722

[10] Lee J, Taneja V, Vassallo R. Cigarette smoking and inflammation: cellular and molecular mechanisms: cellular and molecular mechanisms. J Dent Res. 2012;91:142–9. 10.1177/0022034511421200. 21876032 10.1177/0022034511421200 PMC3261116

[11] Bruijnzeel AW. Tobacco addiction and the dysregulation of brain stress systems. Neurosci Biobehav Rev. 2012;36:1418–41. 10.1016/j.neubiorev.2012.02.015. 22405889 10.1016/j.neubiorev.2012.02.015 PMC3340450

[12] American Psychiatric Association. Diagnostic and statistical manual of mental disorders (DSM-5 (R)). 5th edition. Arlington, TX: American Psychiatric Association Publishing; 2013.

[13] Hofmann SG, Curtiss J, Carpenter JK, Kind S. Effect of treatments for depression on quality of life: a meta-analysis. Cogn Behav Ther. 2017;46:265–86. 10.1080/16506073.2017.1304445. 28440699 10.1080/16506073.2017.1304445 PMC5663193

[14] König H, Rommel A, Thom J, Schmidt C, König H-H, Brettschneider C, et al. The excess costs of depression and the influence of sociodemographic and socioeconomic factors: results from the German health interview and examination survey for adults (DEGS). PharmacoEconomics. 2021;39:667–80. 10.1007/s40273-021-01000-1. 33521892 10.1007/s40273-021-01000-1 PMC8166710

[15] Rowold C, Madia JE. Demographic transitions and lifestyle factors: quantifying the burden of smoking-attributable diseases on Germany’s healthcare system. 2024.

[16] Fluharty M, Taylor AE, Grabski M, Munafò MR. The association of cigarette smoking with depression and anxiety: A systematic review. Nicotine Tob Res. 2017;19:3–13. 10.1093/ntr/ntw140. 27199385 10.1093/ntr/ntw140 PMC5157710

[17] Kock L, Brown J, Cox S, McNeill A, Robson D, Shahab L, et al. Association of psychological distress with smoking cessation, duration of abstinence from smoking, and use of non-combustible nicotine-containing products: A cross-sectional population survey in great Britain. Addict Behav. 2023;138:107570. 10.1016/j.addbeh.2022.107570. 36493683 10.1016/j.addbeh.2022.107570

[18] North C, Marti CN, Loukas A. Longitudinal impact of depressive symptoms and peer tobacco use on the number of tobacco products used by young adults. Int J Environ Res Public Health. 2021;18:11077. 10.3390/ijerph182111077. 34769598 10.3390/ijerph182111077 PMC8582828

[19] Wu Z, Yue Q, Zhao Z, Wen J, Tang L, Zhong Z, et al. A cross-sectional study of smoking and depression among US adults: NHANES (2005–2018). Front Public Health. 2023;11:1081706. 10.3389/fpubh.2023.1081706. 36794066 10.3389/fpubh.2023.1081706 PMC9922891

[20] de Boer N, Vermeulen J, Lin B, van Os J, Ten Have M, de Graaf R, et al. Longitudinal associations between alcohol use, smoking, genetic risk scoring and symptoms of depression in the general population: a prospective 6-year cohort study. Psychol Med. 2023;53:1409–17. 10.1017/S0033291721002968. 35023464 10.1017/S0033291721002968 PMC10009403

[21] Wu AD, Gao M, Aveyard P, Taylor G. Smoking cessation and changes in anxiety and depression in adults with and without psychiatric disorders. JAMA Netw Open. 2023;6. 10.1001/jamanetworkopen.2023.16111. 10.1001/jamanetworkopen.2023.16111 PMC10233414 37256615

[22] Taylor GMJ, Lindson N, Farley A, Leinberger-Jabari A, Sawyer K, te Water Naudé R et al. Smoking cessation for improving mental health. Cochrane Libr. 2021;2021. 10.1002/14651858.cd013522.pub2 10.1002/14651858.CD013522.pub2 PMC8121093 33687070

[23] Farooqui M, Shoaib S, Afaq H, Quadri S, Zaina F, Baig A, et al. Bidirectionality of smoking and depression in adolescents: a systematic review. Trends Psychiatry Psychother. 2023. 10.47626/2237-6089-2021-0429. 35738567 10.47626/2237-6089-2021-0429 PMC10416256

[24] Berger K, Rietschel M, Rujescu D. The value of mega cohorts for psychiatric research. World J Biol Psychiatry. 2023;24:860–4. 10.1080/15622975.2021.2011405. 35302921 10.1080/15622975.2021.2011405

[25] Peters A, German National Cohort (NAKO) Consortium, Peters A, Greiser KH, Göttlicher S, Ahrens W, et al. Framework and baseline examination of the German National cohort (NAKO). Eur J Epidemiol. 2022. 10.1007/s10654-022-00890-5. 36260190 10.1007/s10654-022-00890-5 PMC9581448

[26] Rach S, Sand M, Reineke A, Becher H, Greiser KH, Wolf K, et al. The baseline examinations of the German National cohort (NAKO): recruitment protocol, response, and weighting. Eur J Epidemiol. 2025. 10.1007/s10654-025-01219-8. 40259125 10.1007/s10654-025-01219-8 PMC12145326

[27] Lecrubier Y, Weiller E, Herugeta T, Amorim P, Bonora LI, Lépine JP, et al. Mini international neuropsychiatric interview German version 5.0. 0. München: Psychiatrischen Universitätsklinik München; 1999.

[28] Streit F, Zillich L, Frank J, Kleineidam L, Wagner M, Baune BT, et al. Lifetime and current depression in the German National cohort (NAKO). World J Biol Psychiatry. 2023;24:865–80. 10.1080/15622975.2021.2014152. 34870540 10.1080/15622975.2021.2014152

[29] Kroenke K, Spitzer RL, Williams JBW. The PHQ-9. J Gen Intern Med. 2001;16:606–13. 10.1046/j.1525-1497.2001.016009606.x. 11556941 10.1046/j.1525-1497.2001.016009606.x PMC1495268

[30] UNESCO United Nations Educational, Scientific and Cultural Organization. International standard classification of education, ISCED 1997. Advances in Cross-National comparison. Boston, MA: Springer US; 2003. pp. 195–220. 10.1007/978-1-4419-9186-7_10.

[31] Dragano N, Reuter M, Greiser KH, Becher H, Zeeb H, Mikolajczyk R, et al. Socio-demographic and employment-related factors in the German National cohort (GNC; NAKO Gesundheitsstudie). Bundesgesundheitsblatt Gesundheitsforschung Gesundheitsschutz. 2020;63:267–78. 10.1007/s00103-020-03098-8. 32034444 10.1007/s00103-020-03098-8

[32] Bush K, Kivlahan DR, McDonell MB, Fihn SD, Bradley KA. The AUDIT alcohol consumption questions (AUDIT-C): an effective brief screening test for problem drinking. Ambulatory care quality improvement project (ACQUIP). Alcohol use disorders identification test. Arch Intern Med. 1998;158:1789–95. 10.1001/archinte.158.16.1789. 9738608 10.1001/archinte.158.16.1789

[33] Sutin AR, Terracciano A, Milaneschi Y, An Y, Ferrucci L, Zonderman AB. The trajectory of depressive symptoms across the adult life span. JAMA Psychiatry. 2013;70:803–11. 10.1001/jamapsychiatry.2013.193. 23760442 10.1001/jamapsychiatry.2013.193 PMC3740038

[34] Hiscock R, Bauld L, Amos A, Fidler JA, Munafò M. Socioeconomic status and smoking: a review. Ann N Y Acad Sci. 2012;1248:107–23. 10.1111/j.1749-6632.2011.06202.x. 22092035 10.1111/j.1749-6632.2011.06202.x

[35] Lorant V, Deliège D, Eaton W, Robert A, Philippot P, Ansseau M. Socioeconomic inequalities in depression: a meta-analysis. Am J Epidemiol. 2003;157:98–112. 10.1093/aje/kwf182. 12522017 10.1093/aje/kwf182

[36] Taylor AE, Fluharty ME, Bjørngaard JH, Gabrielsen ME, Skorpen F, Marioni RE, et al. Investigating the possible causal association of smoking with depression and anxiety using Mendelian randomisation meta-analysis: the CARTA consortium. BMJ Open. 2014;4:e006141. 10.1136/bmjopen-2014-006141. 25293386 10.1136/bmjopen-2014-006141 PMC4187451

[37] Wang P, Abdin E, Asharani PV, Seet V, Devi F, Roystonn K, et al. Nicotine dependence in patients with major depressive disorder and psychotic disorders and its relationship with quality of life. Int J Environ Res Public Health. 2021;18:13035. 10.3390/ijerph182413035. 34948665 10.3390/ijerph182413035 PMC8701186

[38] Jamal M, Van der Willem AJ, Cuijpers P, Penninx BWJH. Association of smoking and nicotine dependence with severity and course of symptoms in patients with depressive or anxiety disorder. Drug Alcohol Depend. 2012;126:138–46. 10.1016/j.drugalcdep.2012.05.001. 22633368 10.1016/j.drugalcdep.2012.05.001

[39] Yun W-J, Shin M-H, Kweon S-S, Ryu S-Y, Rhee J-A. Association of smoking status, cumulative smoking, duration of smoking cessation, age of starting smoking, and depression in Korean adults. BMC Public Health. 2012;12:724. 10.1186/1471-2458-12-724. 22938088 10.1186/1471-2458-12-724 PMC3495214

[40] Park SK, Oh C-M, Kim E, Ryoo J-H, Jung JY. The longitudinal analysis for the association between smoking and the risk of depressive symptoms. BMC Psychiatry. 2024;24. 10.1186/s12888-024-05828-7. 10.1186/s12888-024-05828-7 PMC11094926 38750466

[41] Devkota B, Salas J, Garfield L. Increased risk of major depression with early age of exposure to cigarettes. Am J Prev Med. 2016;51:933–8. 10.1016/j.amepre.2016.05.022. 27436333 10.1016/j.amepre.2016.05.022

[42] Taylor G, Mcneill A, Girling A, Farley A, Lindson-Hawley N, Aveyard P. Change in mental health after smoking cessation: systematic review and meta-analysis BMJ. BMJ. 2014;348. 10.1136/bmj.g1151. 10.1136/bmj.g1151 PMC3923980 24524926

[43] Rodríguez-Cano R, López-Durán A, del Río EF, Martínez-Vispo C, Martínez Ú, Becoña E. Smoking cessation and depressive symptoms at 1-, 3-, 6-, and 12-months follow-up. J Affect Disord. 2016;191:94–9. 10.1016/j.jad.2015.11.042. 26655118 10.1016/j.jad.2015.11.042

[44] Fedak KM, Bernal A, Capshaw ZA, Gross S. Applying the Bradford hill criteria in the 21st century: how data integration has changed causal inference in molecular epidemiology. Emerg Themes Epidemiol. 2015;12:14. 10.1186/s12982-015-0037-4. 26425136 10.1186/s12982-015-0037-4 PMC4589117

[45] Hitsman B, Papandonatos GD, McChargue DE, DeMott A, Herrera MJ, Spring B, et al. Past major depression and smoking cessation outcome: a systematic review and meta-analysis update. Addiction. 2013;108:294–306. 10.1111/add.12009. 23072580 10.1111/add.12009 PMC3593055

[46] Yao Y, Xu Y, Cai Z, Liu Q, Ma Y, Li AN, et al. Determination of shared genetic etiology and possible causal relations between tobacco smoking and depression. Psychol Med. 2021;51:1870–9. 10.1017/S003329172000063X. 32249730 10.1017/S003329172000063X

[47] Moylan S, Jacka FN, Pasco JA, Berk M. How cigarette smoking May increase the risk of anxiety symptoms and anxiety disorders: a critical review of biological pathways. Brain Behav. 2013;3:302–26. 10.1002/brb3.137. 23785661 10.1002/brb3.137 PMC3683289

[48] Bamberg F, Schlett CL, Caspers S, Ringhof S, Günther M, Hirsch JG, et al. Baseline MRI examination in the NAKO health Study—findings on feasibility, participation and dropout rates, comfort, and image quality. Dtsch Arztebl Int. 2024;121:587–93. 10.3238/arztebl.m2024.0151. 39158357 10.3238/arztebl.m2024.0151 PMC11661488

