# Supplementary material for: From cigarettes to symptoms: the association between smoking and depression in the German National Cohort (NAKO)
Source: BMC Public Health. 2025 Dec 19;26:301. doi: 10.1186/s12889-025-25959-0 (PMC12831396; doi:10.1186/s12889-025-25959-0)

**Supplemental figures**


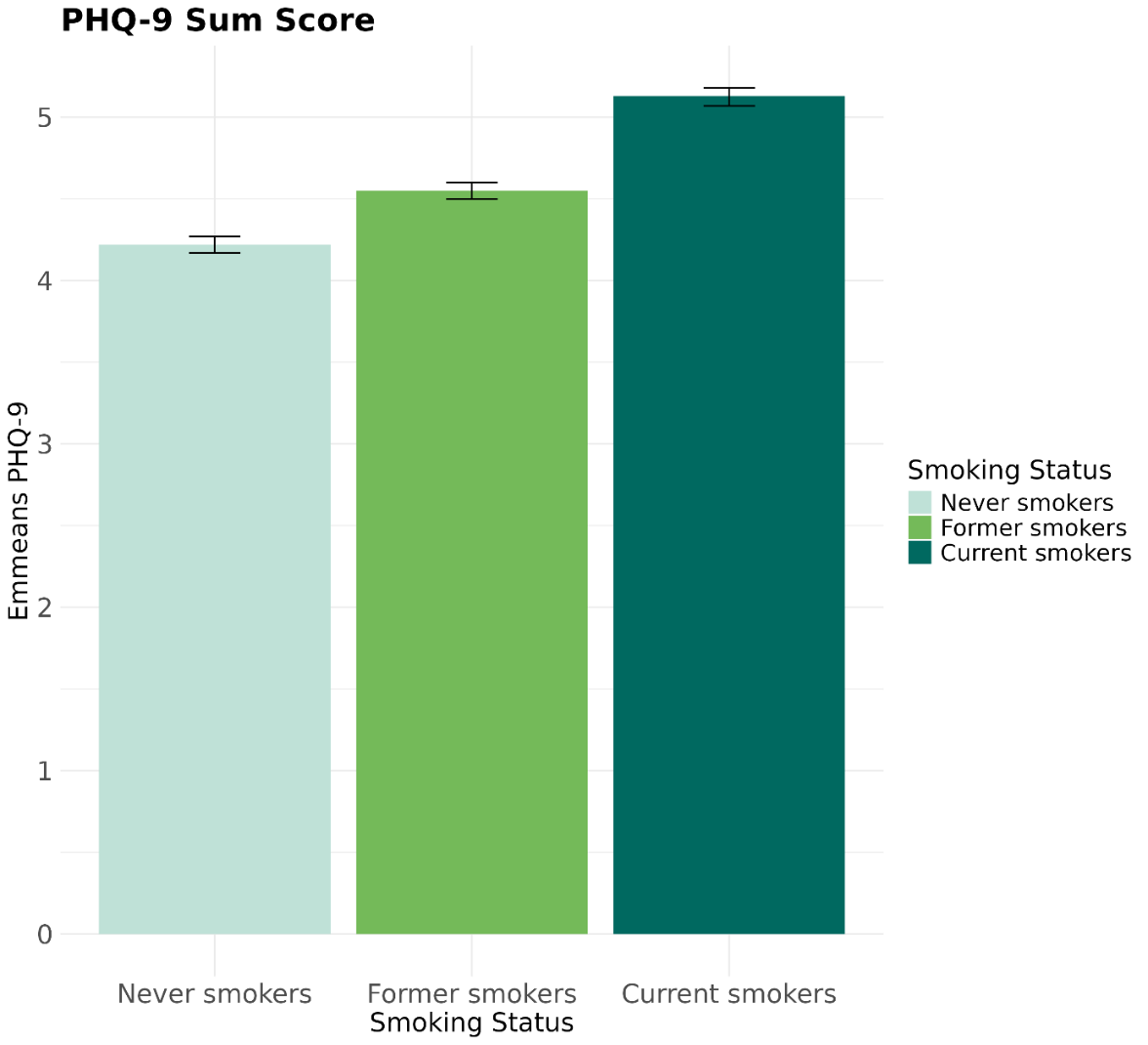
**S1.** Estimated Marginal Means for PHQ-9 sum score by Smoking Status


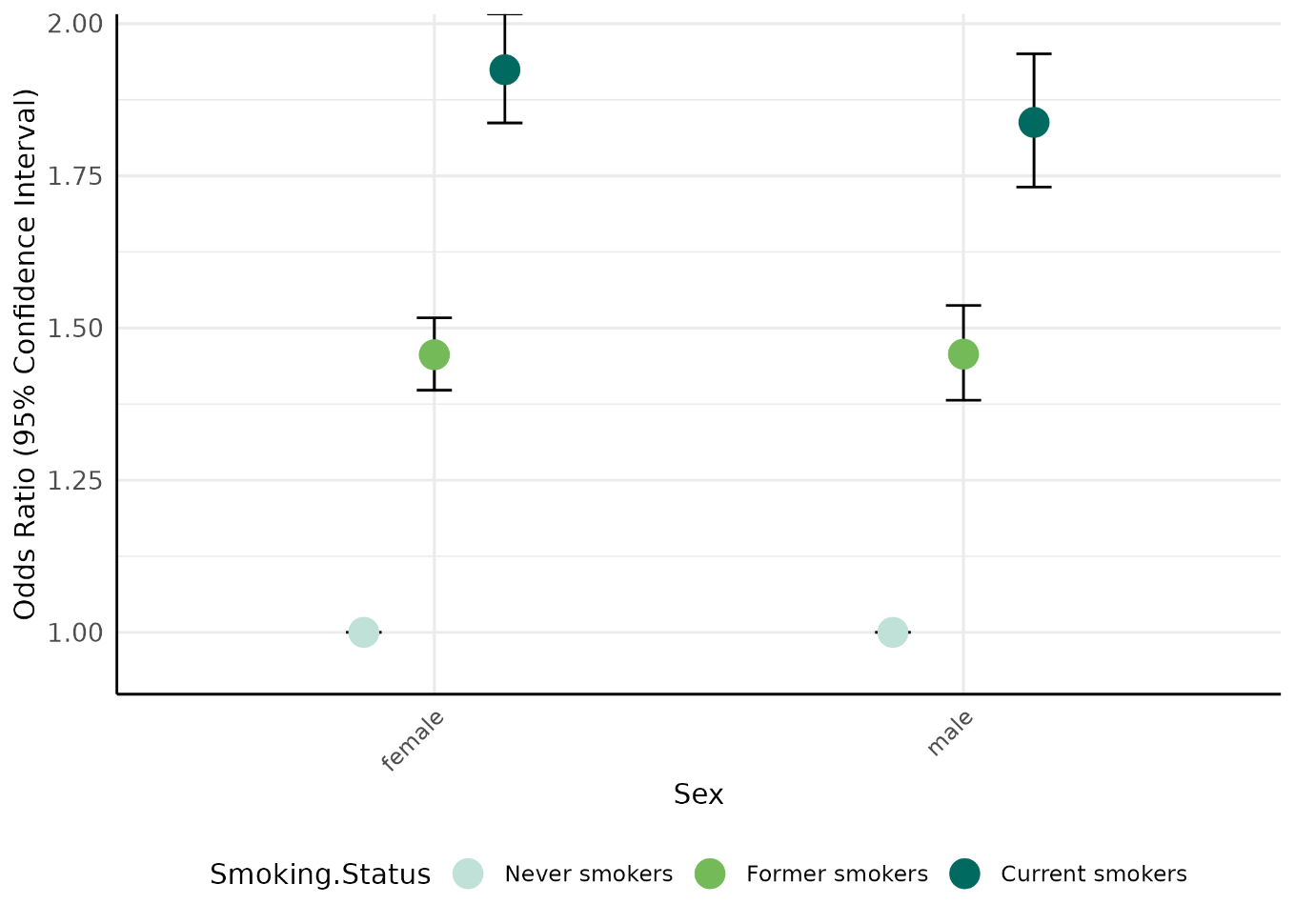
 **S2.** Odds Ratios of Physician’s Diagnosis by Smoking Status Stratified by Sex

**S3.** Odds Ratios of MINI Classification by Smoking Status Stratified by Sex

**
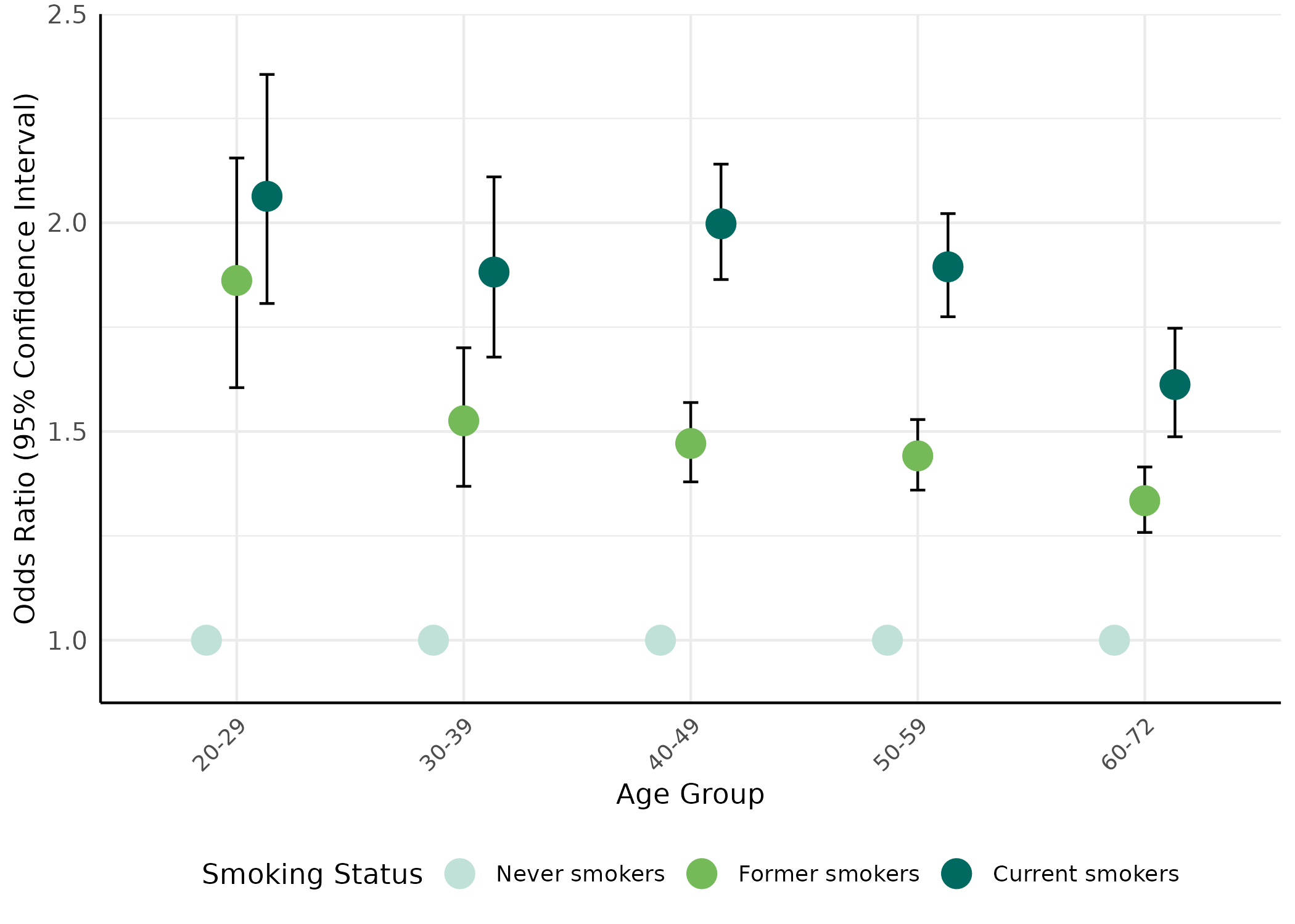

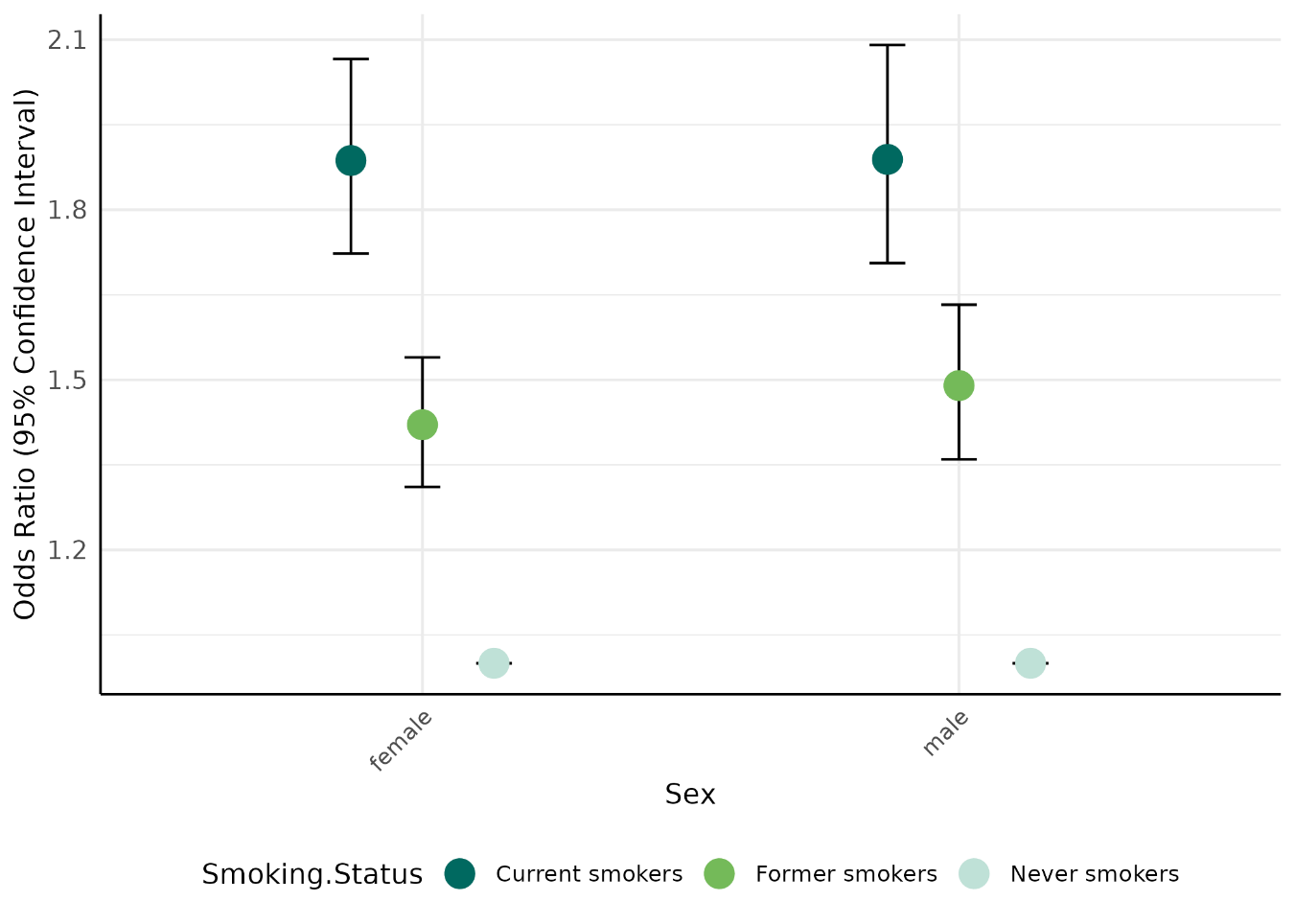
S4.** Odds Ratios of Physician’s Diagnosis by Smoking Status Stratified by Age Group


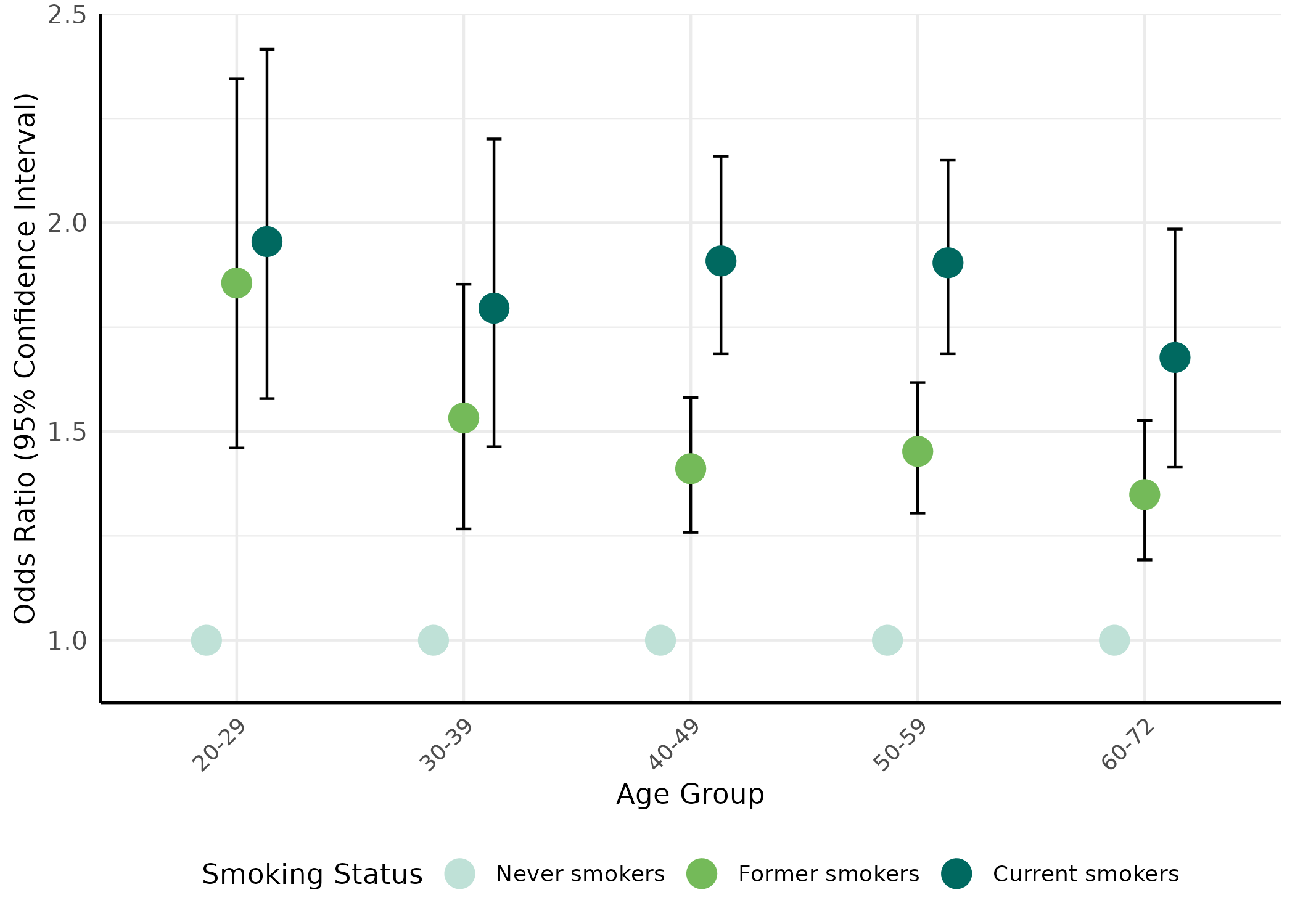
**S5.** Odds Ratios of MINI classification by Smoking Status Stratified by Age Group

**
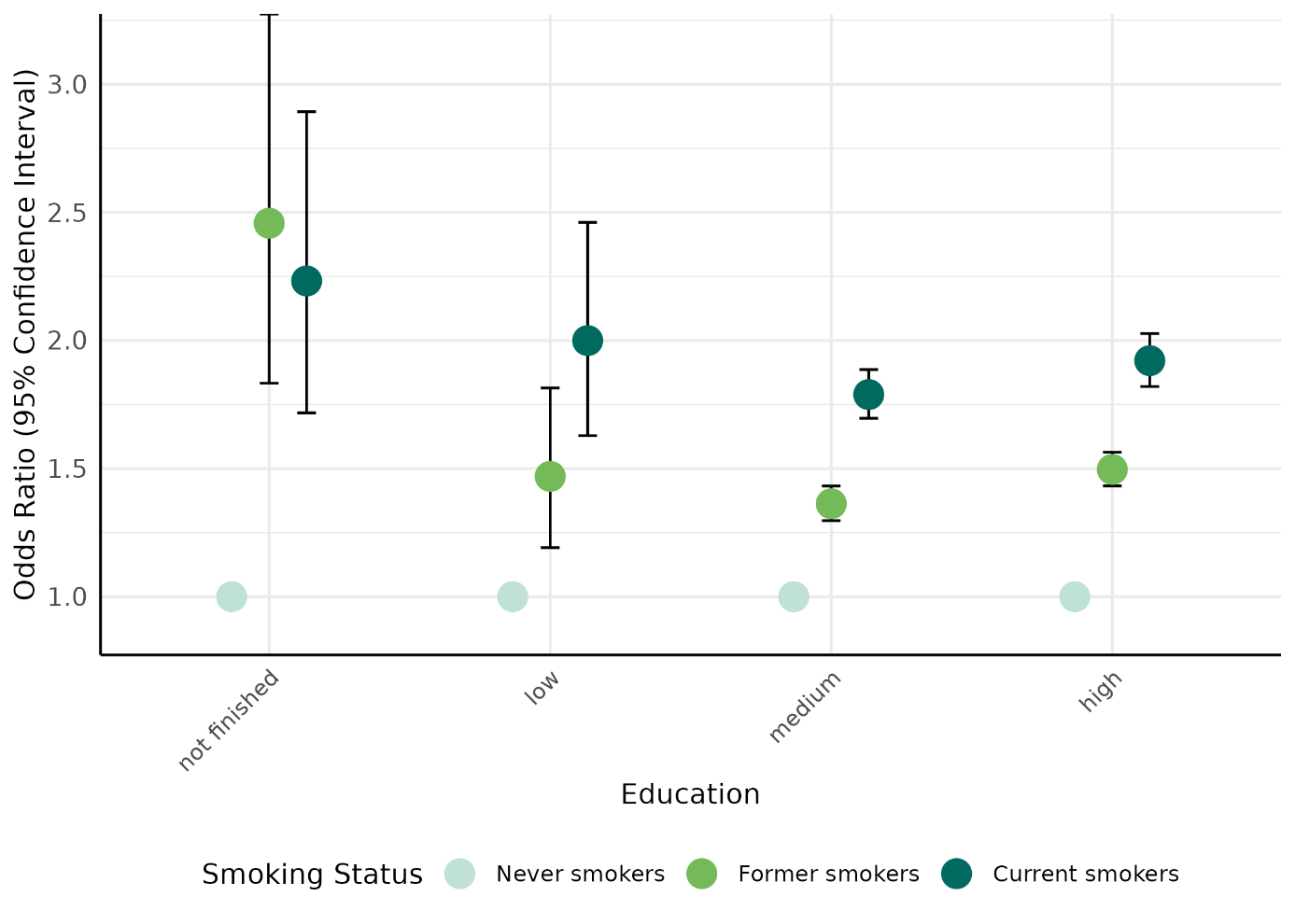
S6.** Odds Ratios of Physician’s Diagnosis by Smoking Status Stratified by Education Level

**
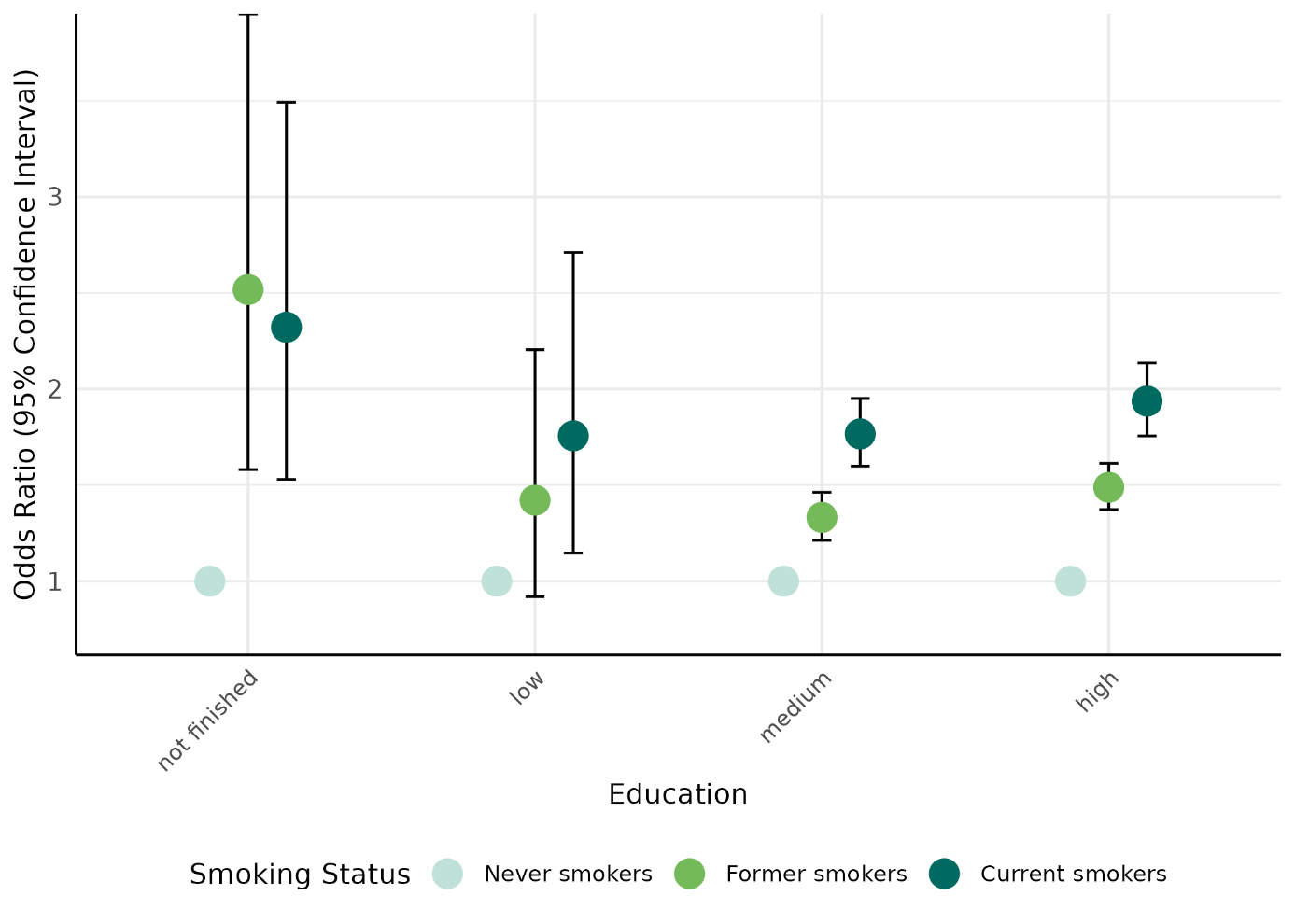
S7.** Odds Ratios of MINI classification by Smoking Status Stratified by Education Level


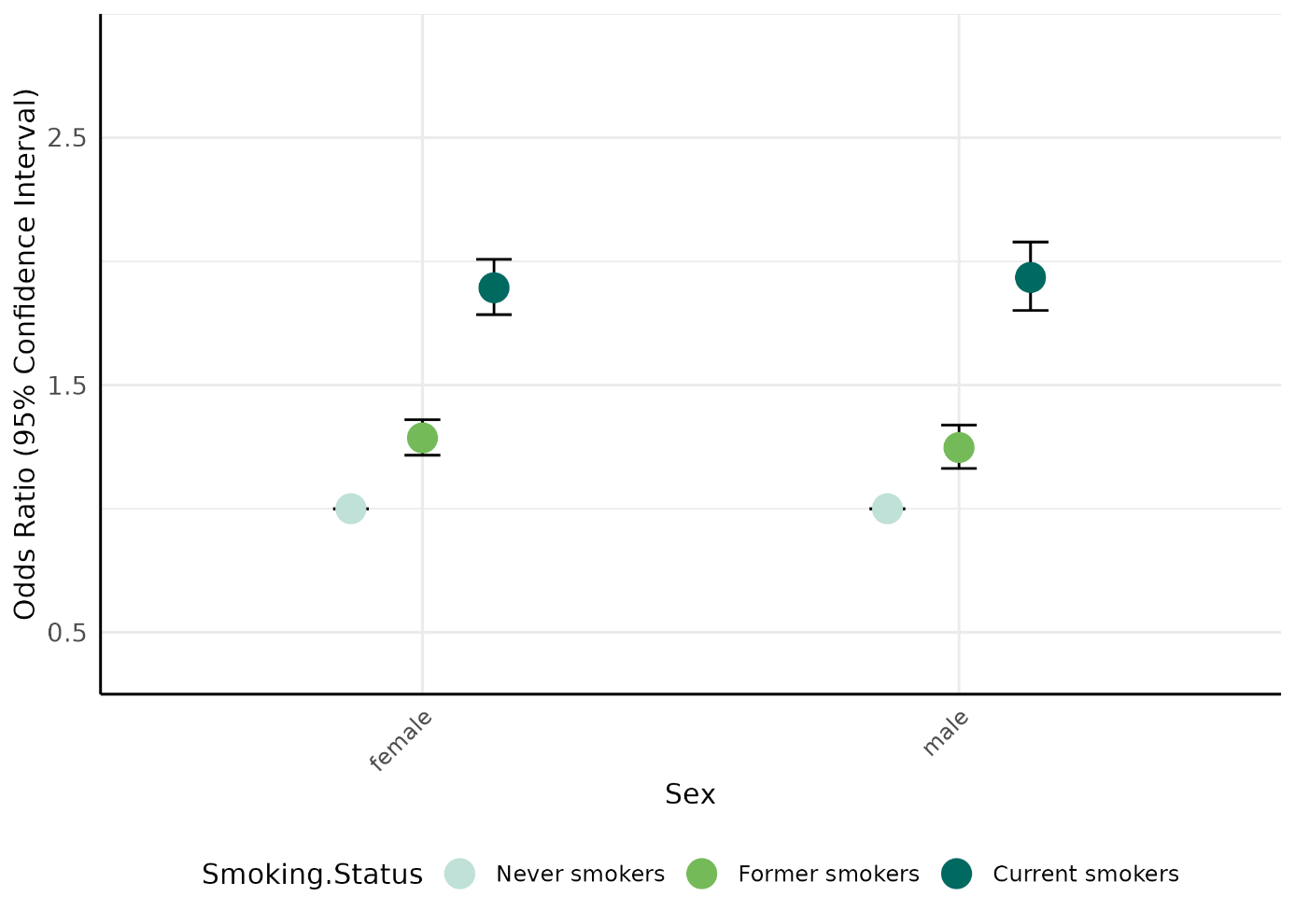
**S8.** Odds Ratios of PHQ-9 cut-off ≥ 10 by Smoking Status Stratified by Sex

**S9.** Odds Ratios of PHQ-9 cut-off ≥ 10 by Smoking Status Stratified by Age Group


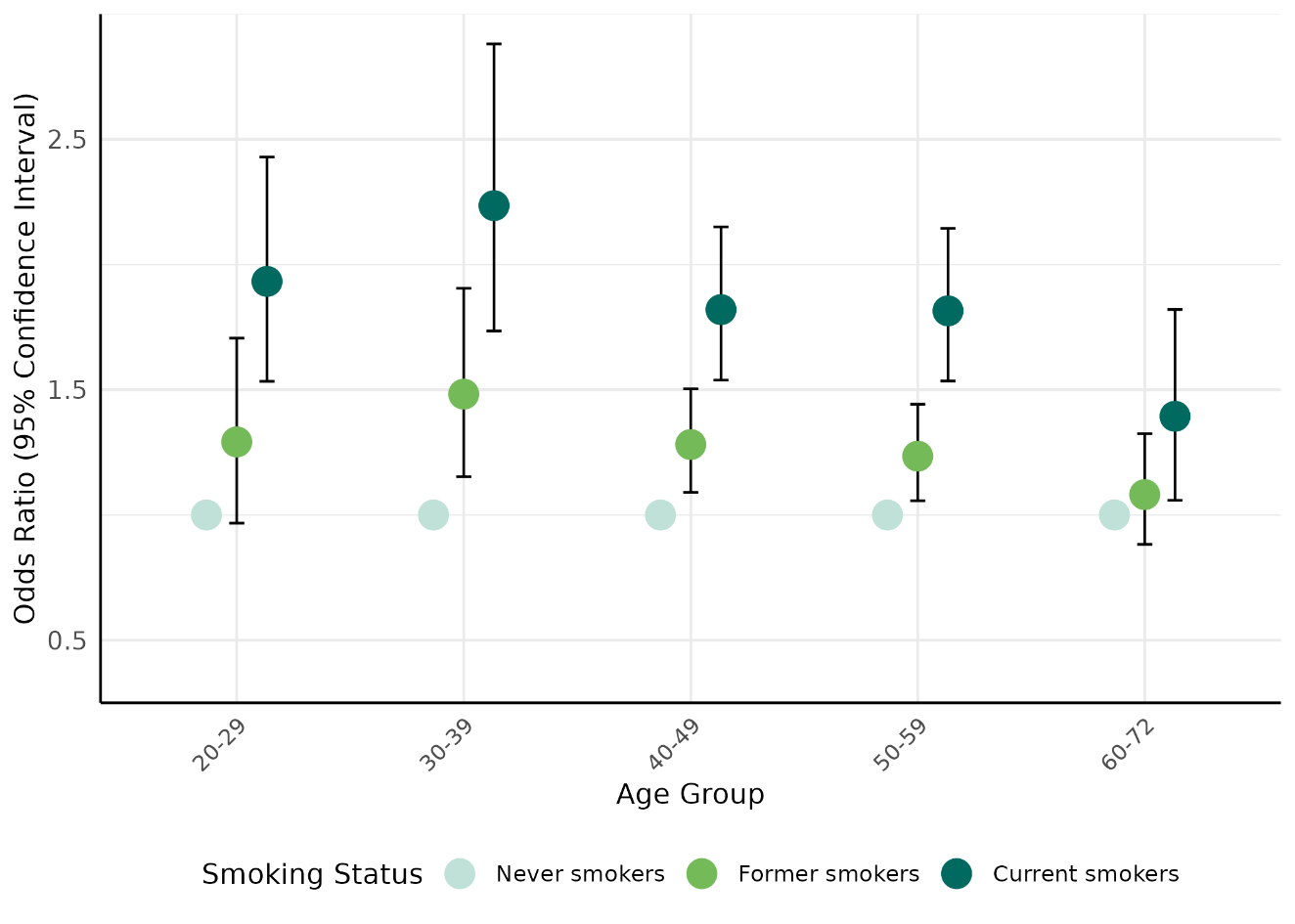


**S10.** Odds Ratios of PHQ-9 cut-off ≥ 10 by Smoking Status Stratified by Education Level


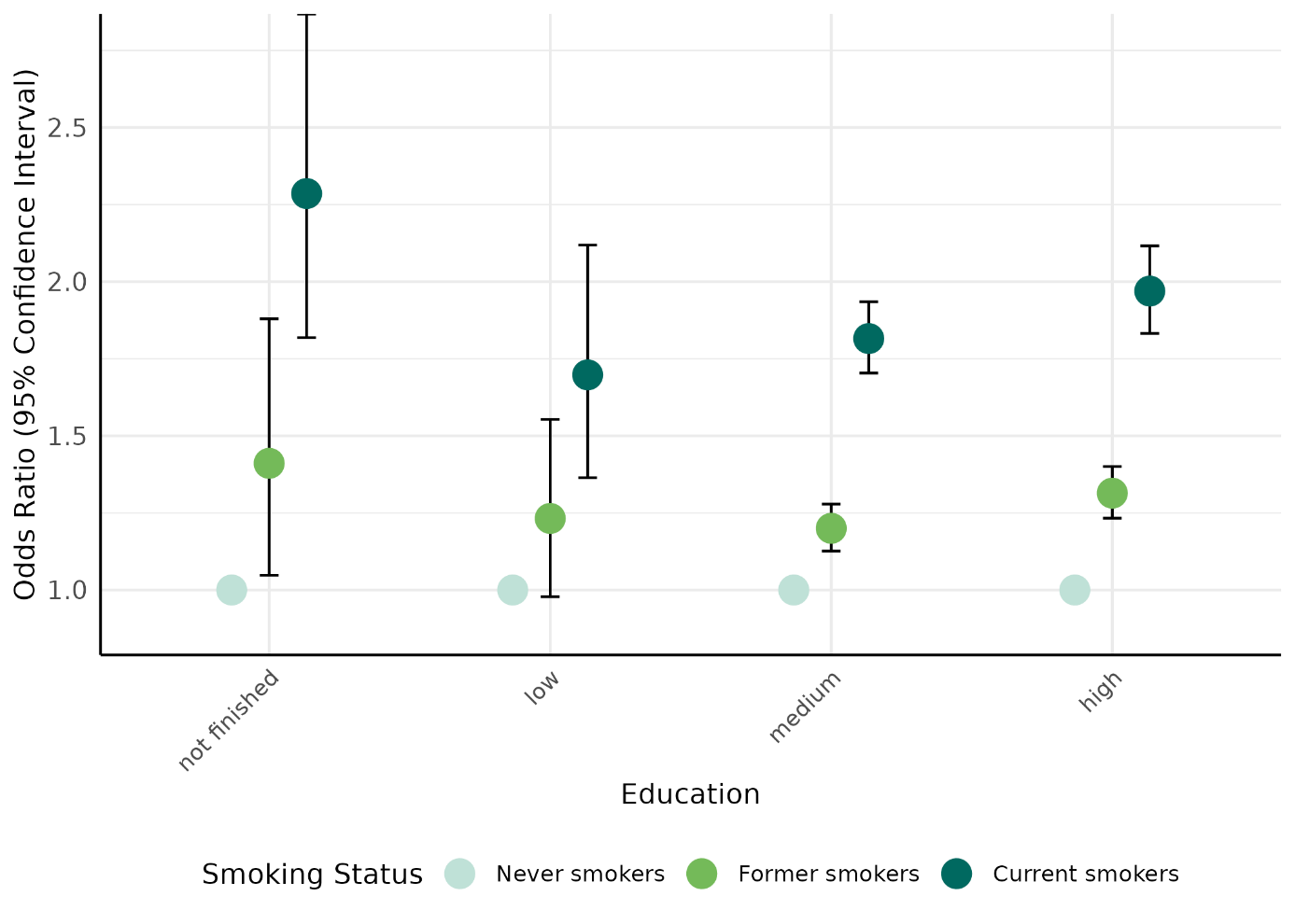


**S11.** Beta Estimates of PHQ-9 Sum Scores by Cigarettes per Day Stratified by Sex
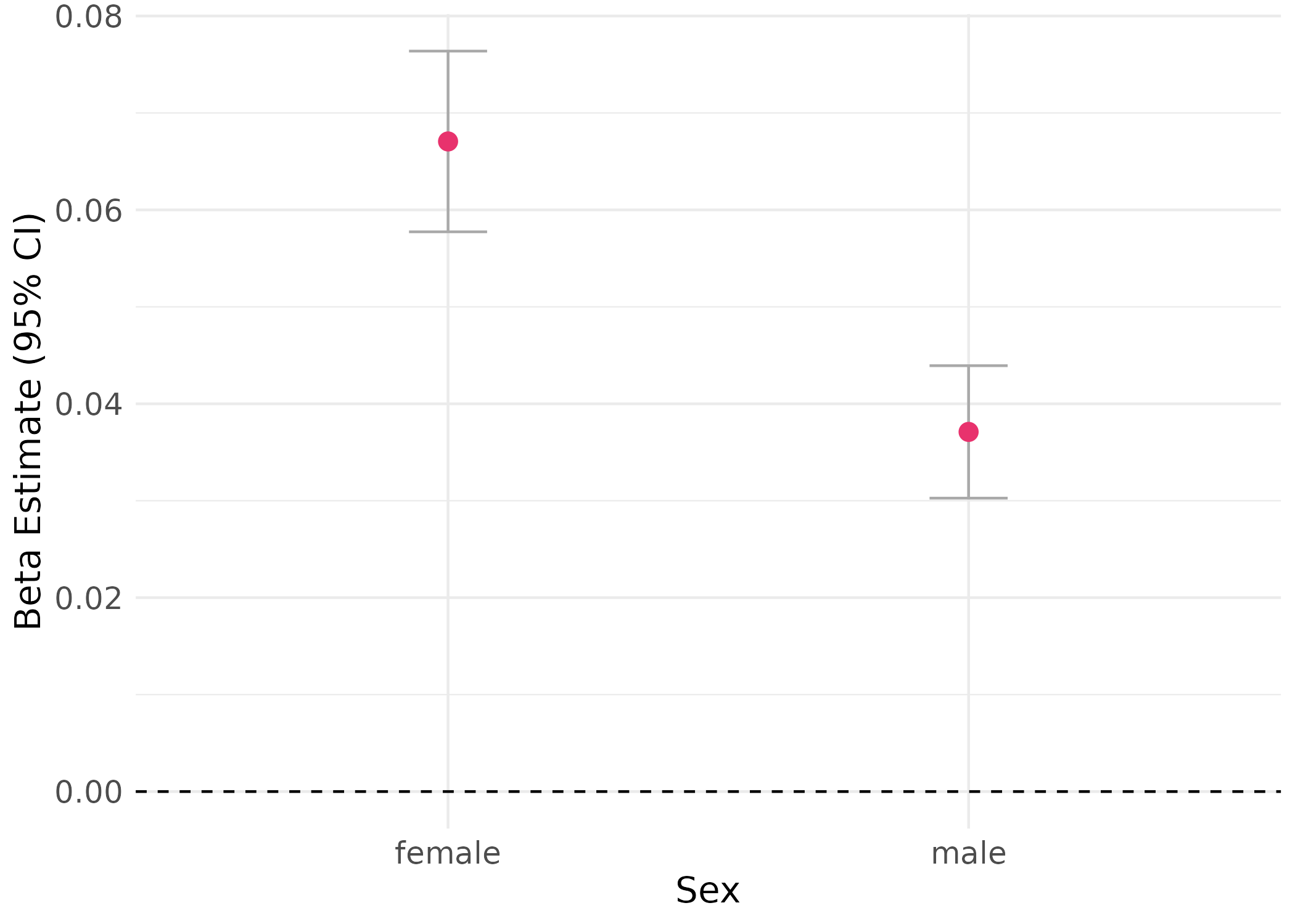


**S12.** Beta Estimates of PHQ-9 Sum Scores by Cigarettes per Day Stratified by Age Group
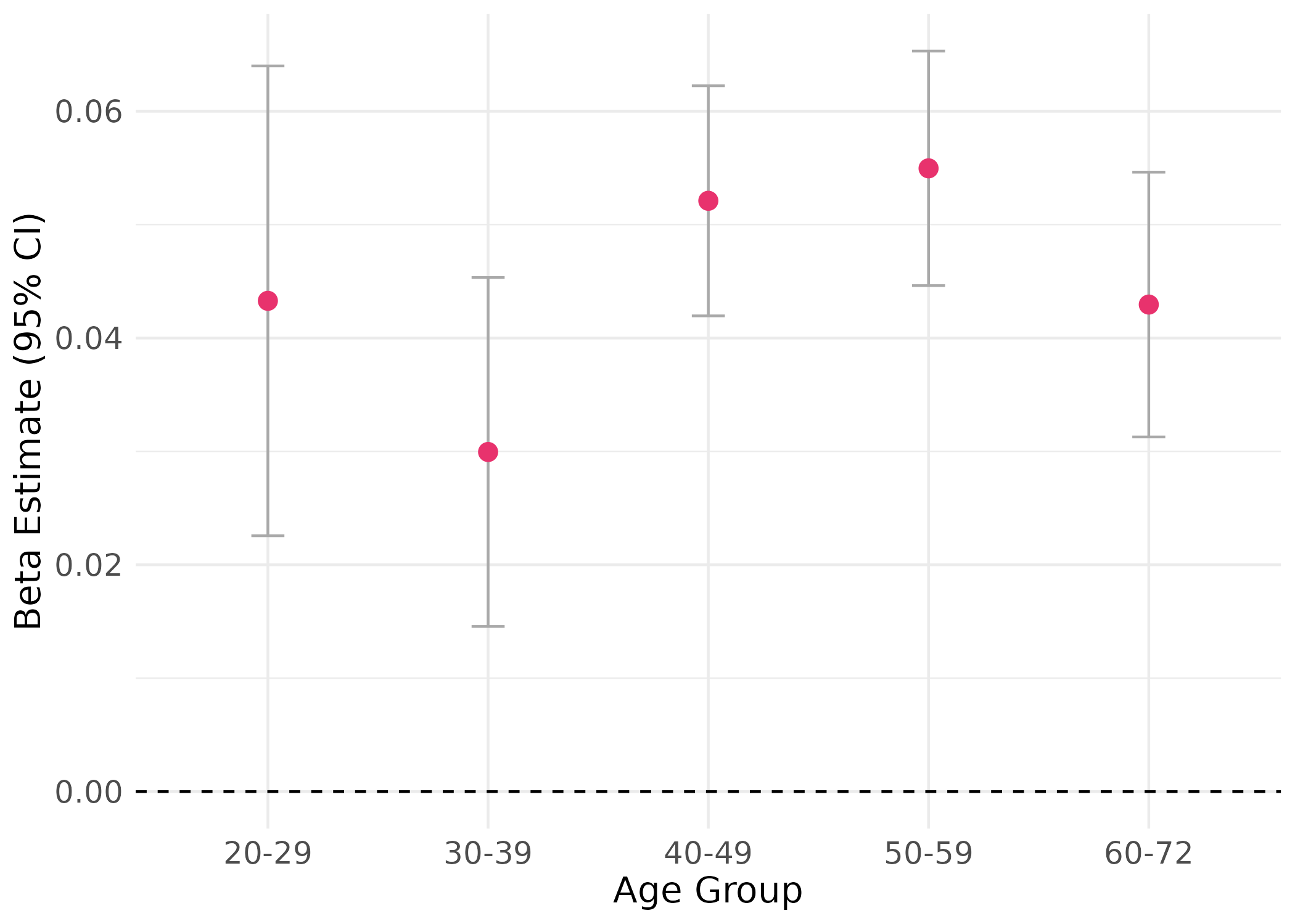


**S13.** Beta Estimates of PHQ-9 Sum Scores by Cigarettes per Day Stratified by Education Level


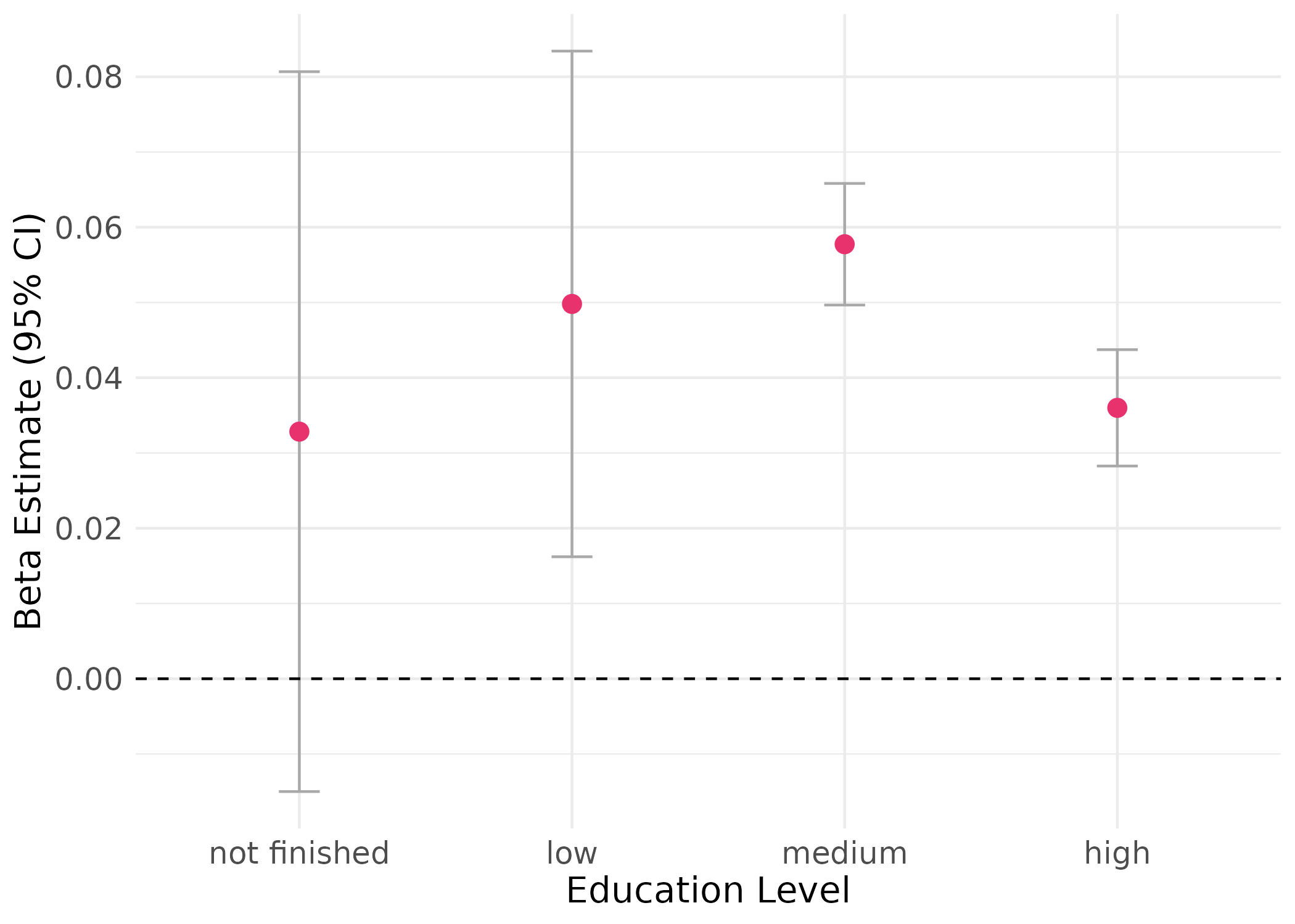


**S14.** Beta Estimates of Age at Depression Onset by Age at Smoking Initiation Stratified by Sex


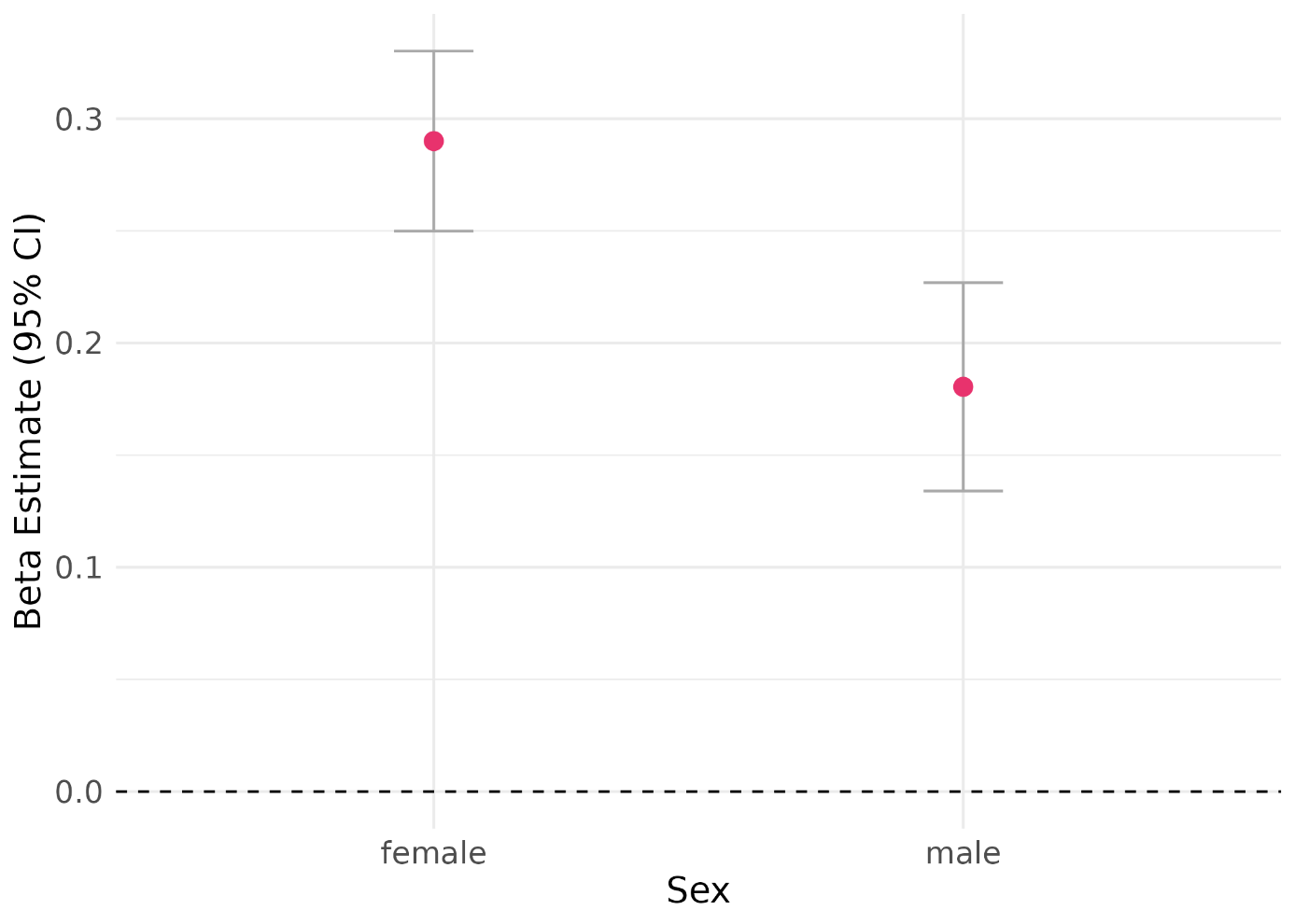


**
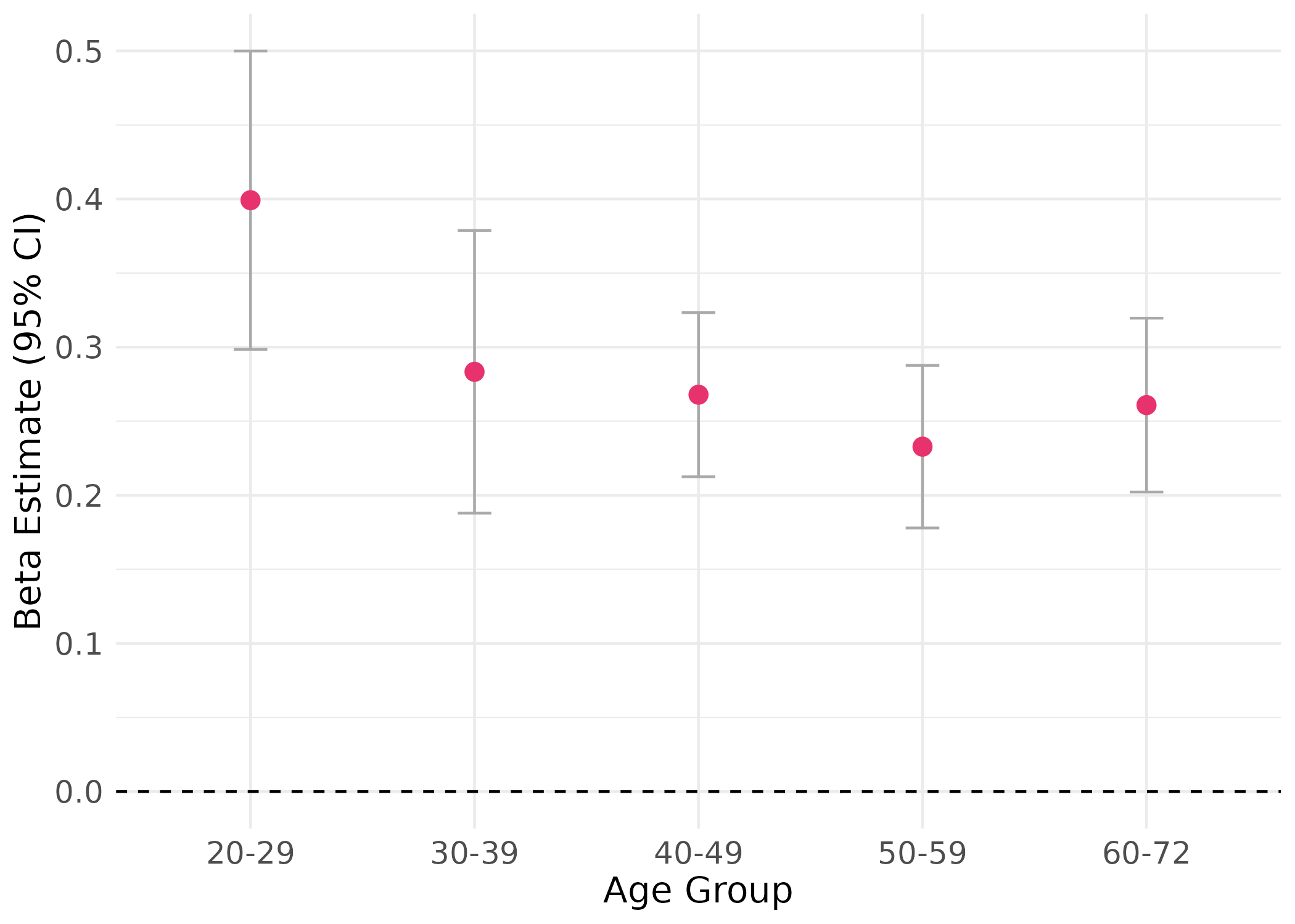
S15.** Beta Estimates of Age at Depression Onset by Age at Smoking Initiation Stratified by Age Group


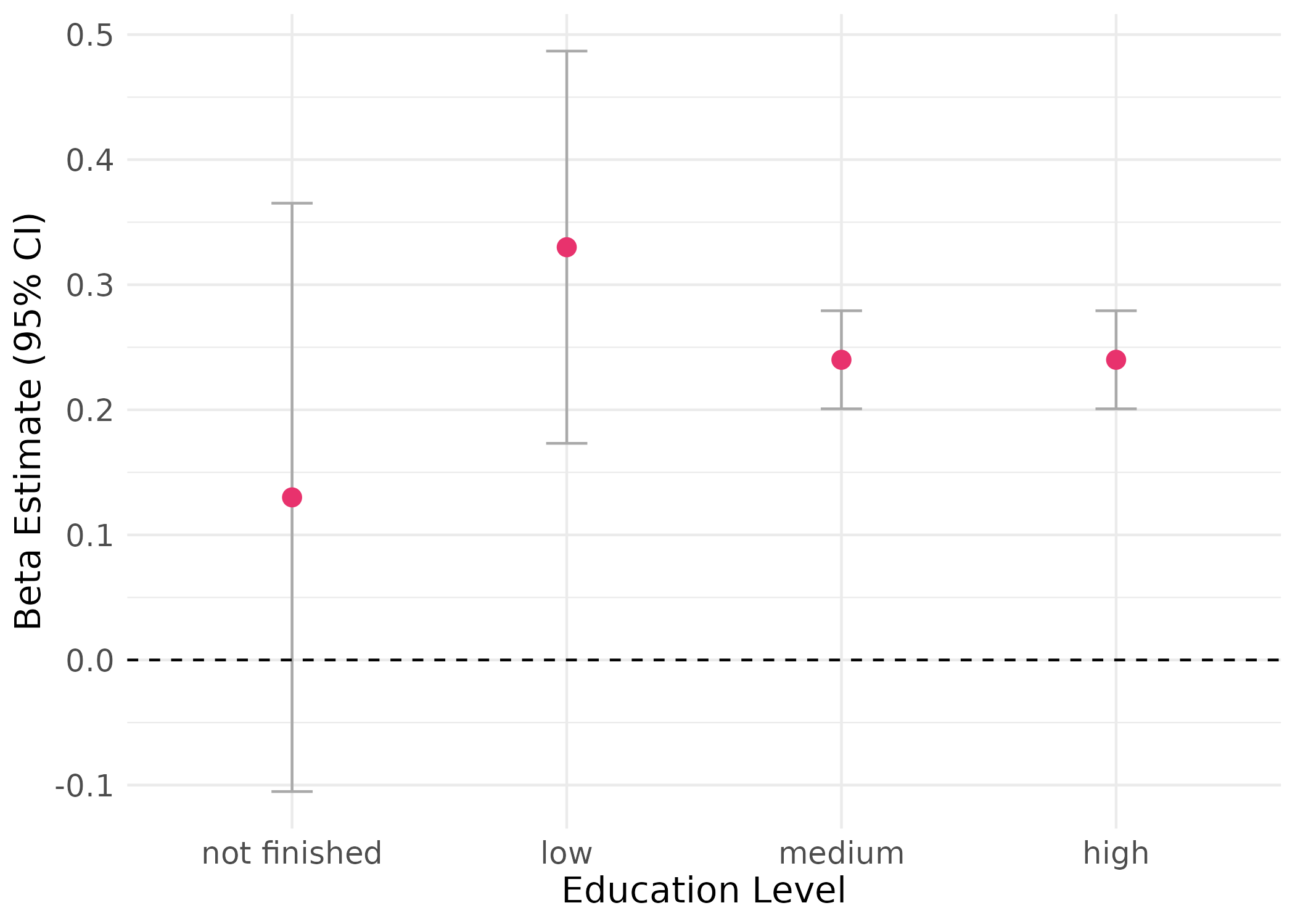
**S16.** Beta Estimates of Age at Depression Onset by Age at Smoking Initiation Stratified by Education Level

**S17.** Beta Estimates of Time since Last Depressive Episode by Time since Smoking Cessation Stratified by Sex


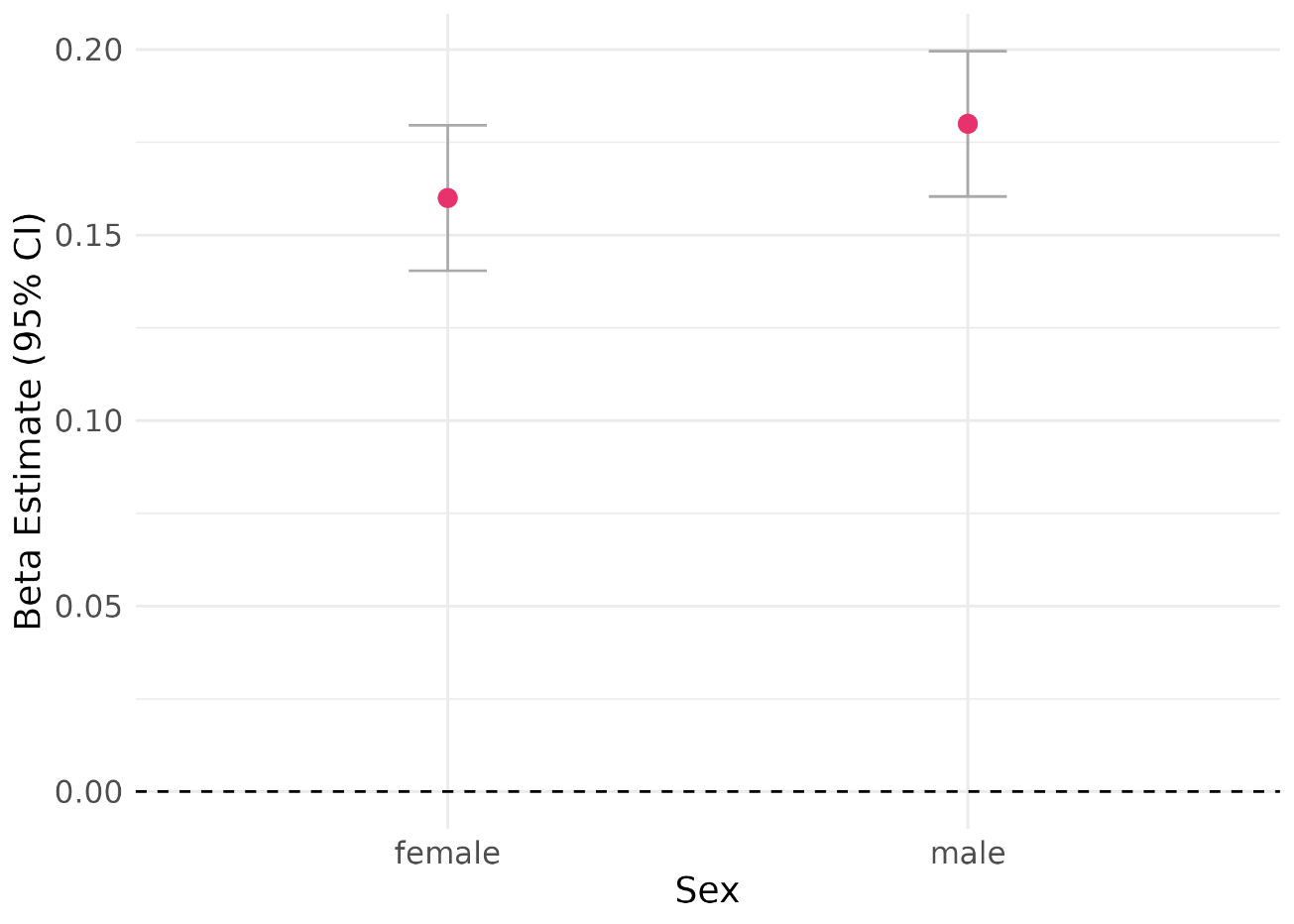


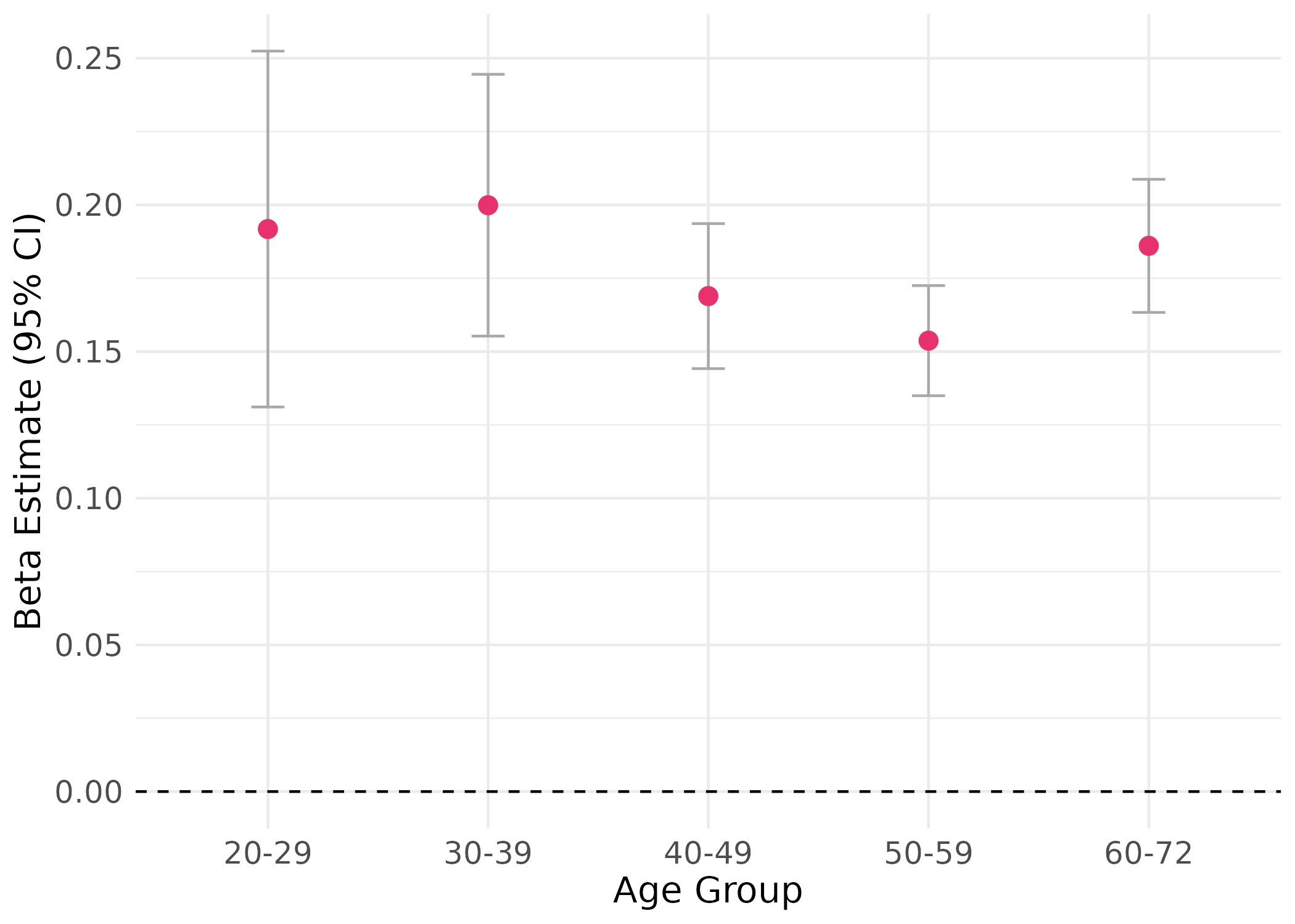
**S18.** Beta Estimates of Time since Last Depressive Episode by Time since Smoking Cessation Stratified by Age Group

**S19.** Beta Estimates of Time since Last Depressive Episode by Time since Smoking Cessation Stratified by Education Level


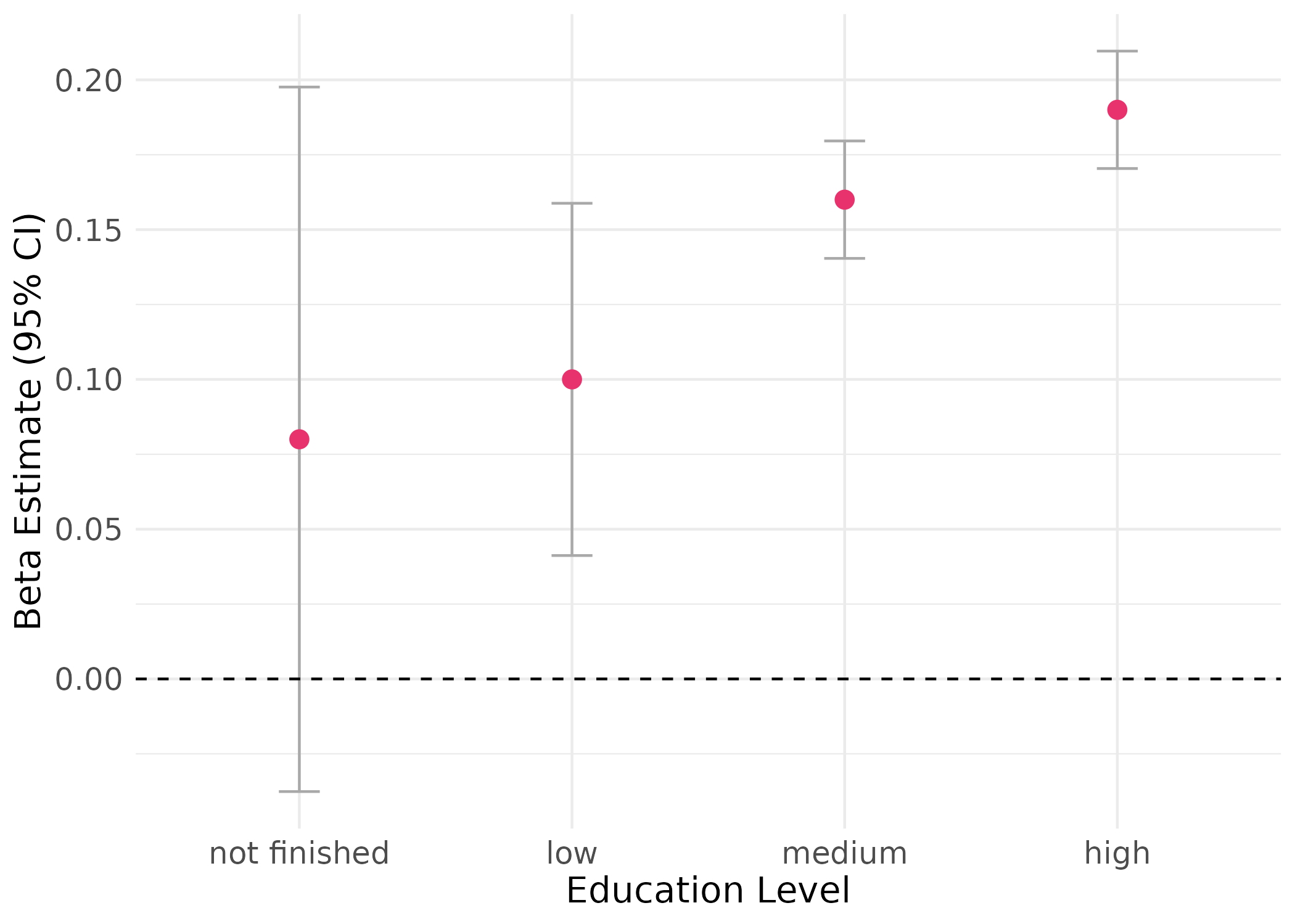


**S20.** Beta Estimates of PHQ-9 Sum Scores by Time since Smoking Cessation Stratified by Age Group


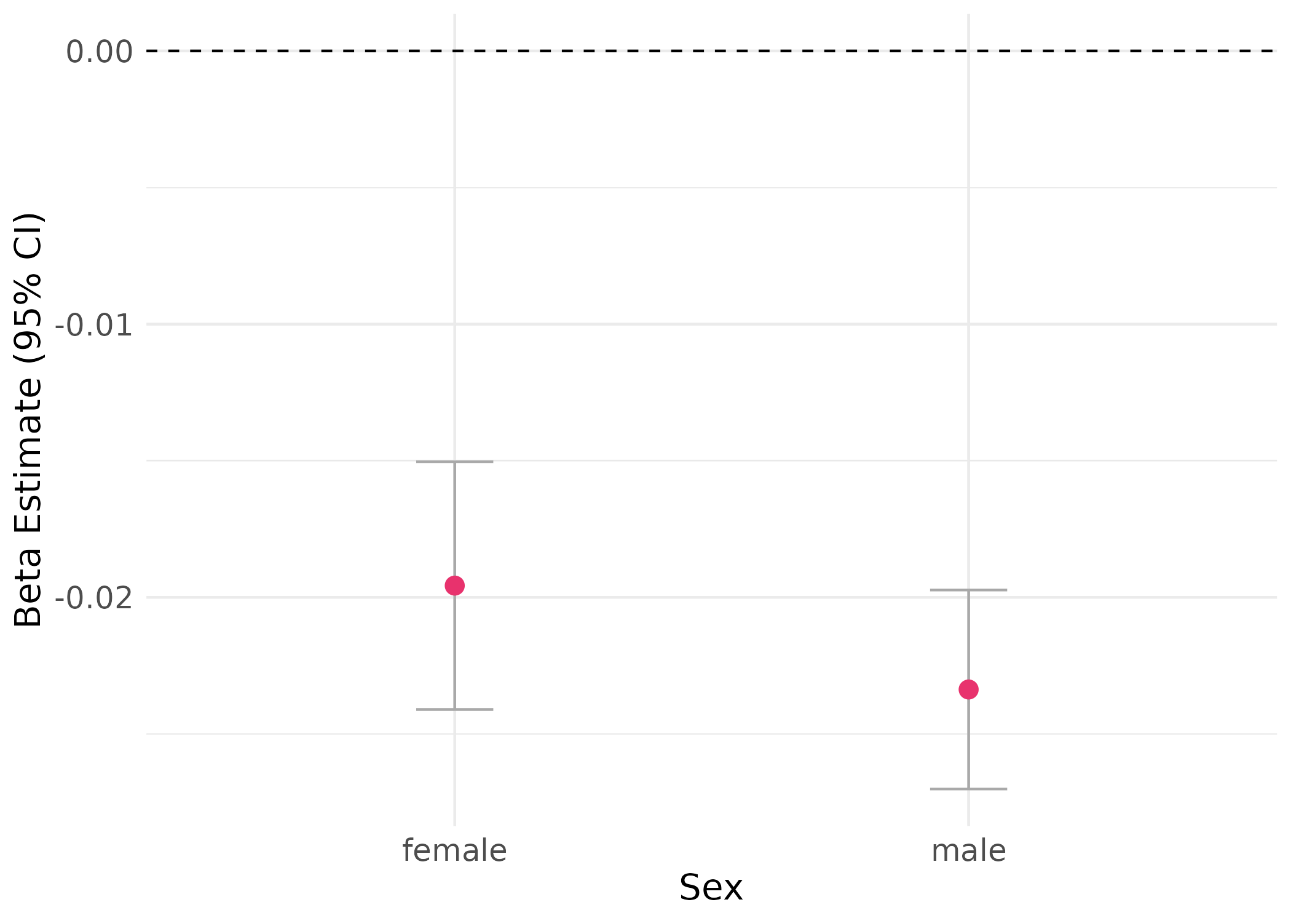


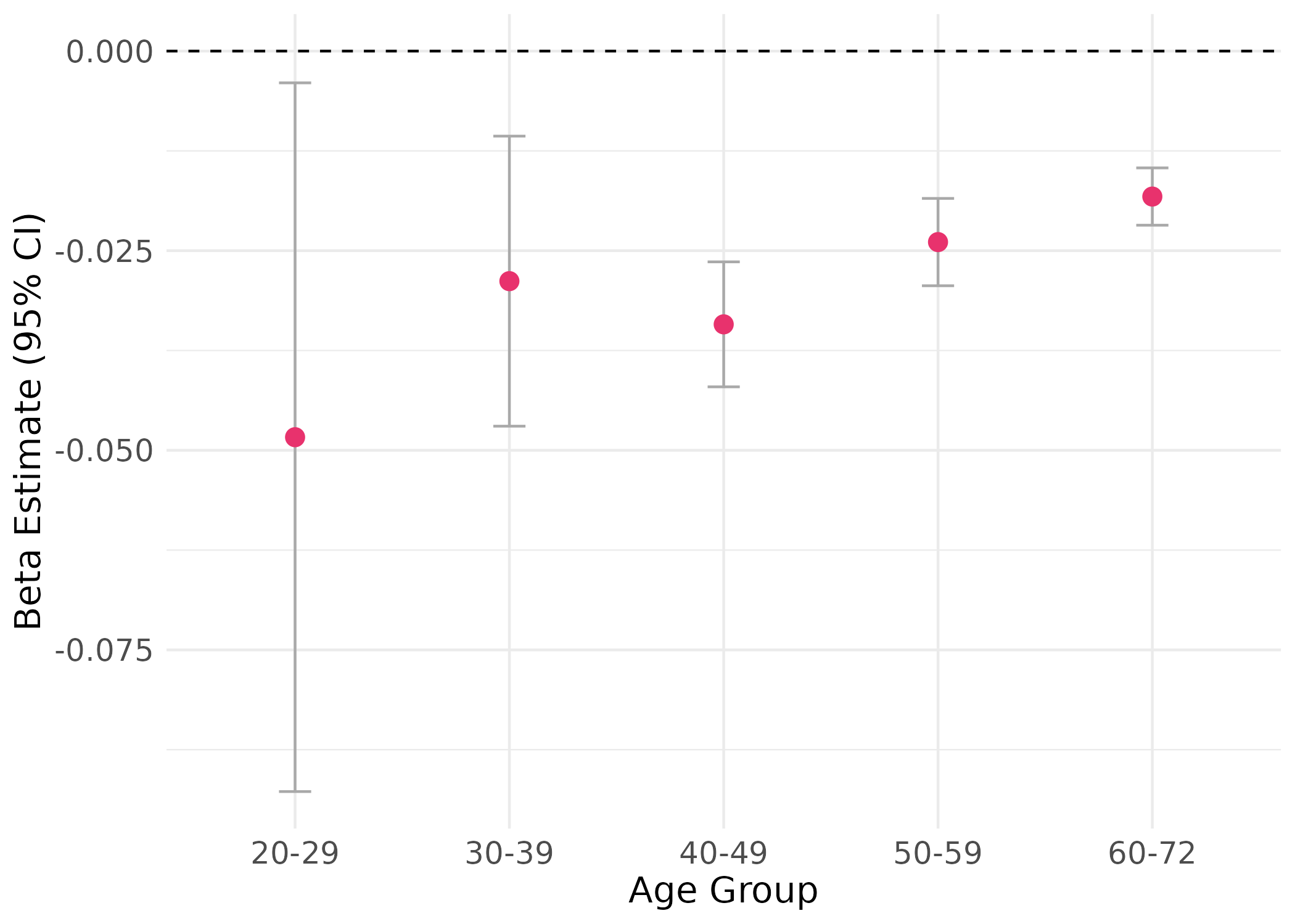
**S21.** Beta Estimates of PHQ-9 Sum Scores by Time since Smoking Cessation Stratified by Age Group

**S22.** Beta Estimates of PHQ-9 Sum Scores by Time since Smoking Cessation Stratified by Education Level


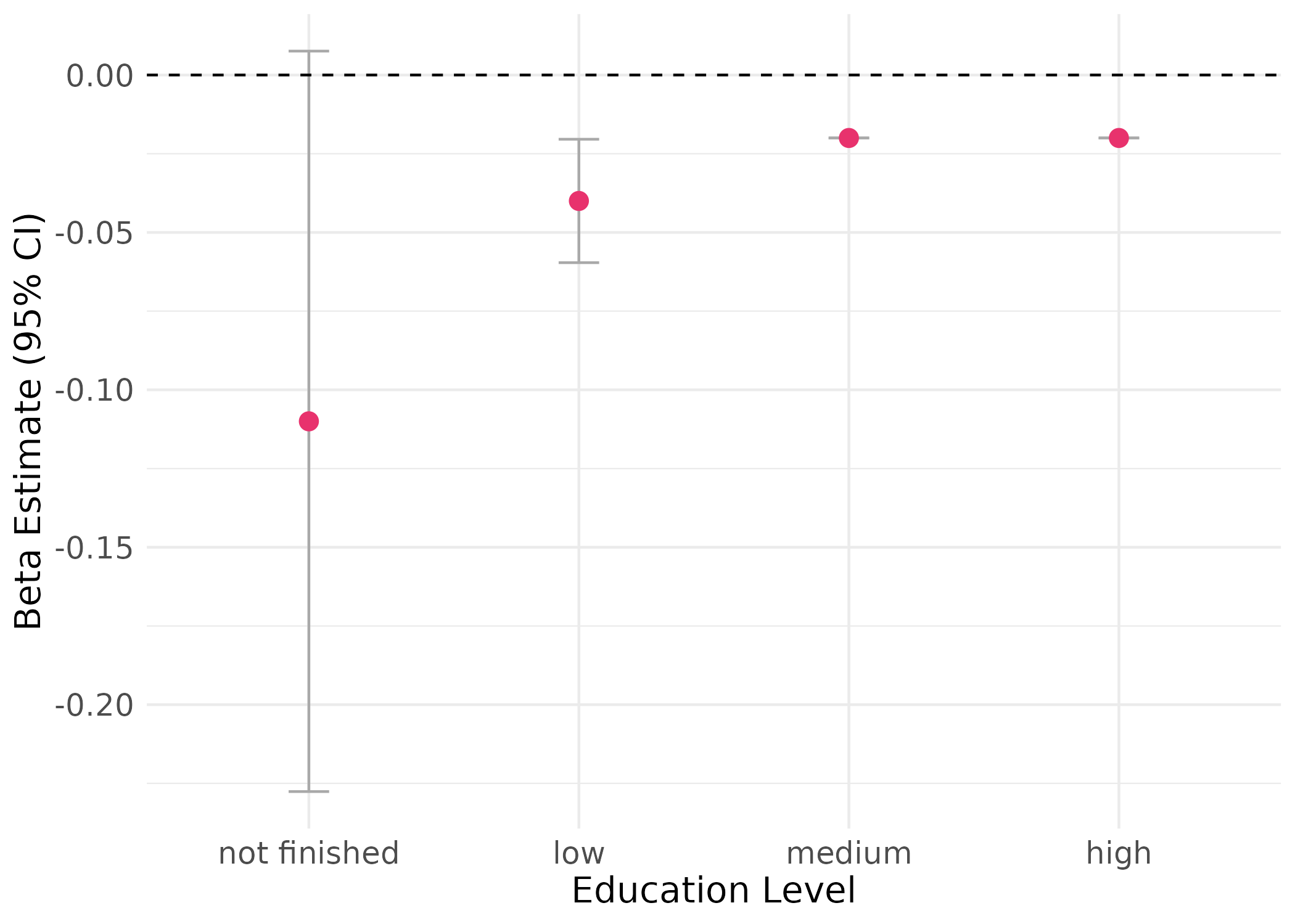

Supplement: Supplementary file 1 — Supplementary Material 1. [file 12889_2025_25959_MOESM1_ESM.docx]
