# Supplementary material for: From cigarettes to symptoms: the association between smoking and depression in the German National Cohort (NAKO)
Source: BMC Public Health. 2025 Dec 19;26:301. doi: 10.1186/s12889-025-25959-0 (PMC12831396; doi:10.1186/s12889-025-25959-0)
Supplement: Supplementary file 2 — Supplementary Material 2. [file 12889_2025_25959_MOESM2_ESM.docx]

**S1 Regression models for all main analyses**

1. *Association of smoking status with lifetime and current depression*

Lifetime depression: glm(physicians_diagnosis ~ smoking_status + age + age² + sex + education_level + BMI + alcohol_consumption)

Lifetime depression L2: glm(MINI_classification ~ smoking_status + age + age² + sex + education_level + BMI + alcohol_consumption)

Current depressive symptoms: glm(PHQ-9_cutoff_score ~ smoking_status + age + age² + sex + education_level + BMI + alcohol_consumption)

Lifetime & current depression: multinom(physicians_diagnosis & PHQ-9_cutoff_score ~ smoking_status + age + age² + sex + education_level + BMI + alcohol_consumption)

1. *Association of cigarettes per day with current depressive symptoms*

lm(PHQ-9_sum_score ~ cigarettes_per_day + age + age² + sex + education_level + BMI + alcohol_consumption)

1. *Association of age at smoking initiation with age at depression onset*

lm(age_at_depression_onset ~ age_at_smoking_initiation + age + age² + sex + education_level + BMI + alcohol_consumption)

1. *Association of time since smoking cessation with time since the last depressive episode*

lm(time_since_the_last_depressive_episode ~ time_since_smoking_cessation + age + age² + sex + education_level + BMI + alcohol_consumption)

1. *Association of time since smoking cessation with current depressive symptoms*

lm(PHQ-9_sum_score ~ time_since_smoking_cessation + age + age² + sex + education_level + BMI + alcohol_consumption)

|  |  | Never smoked | Formerly smoked | | Currently smoking | |  |
| --- | --- | --- | --- | --- | --- | --- | --- |
|  |  | Frequency | Frequency | OR | Frequency | OR | |
| Physician’s diagnosis |  | 11.4% [10.6%,  12.3%] | 15.8%  [14.7%,  16.9%] | 1.46* [1.41,1.50] | 19.6%  [18.3%,  20.9%] | 1.89* [1.83,1.97] | |
| MINI classification |  | 11.6%  [9.9%,  13.5%] | 15.9%  [14.0%,  18.4%] | 1.45* [1.36,1.54] | 19.8%  [17.2%,  22.8%] | 1.89* [1.77,2.02] | |
| PHQ-9 cutoff score |  | 10.5%  [9.7%,  11.5%] | 13.0%  [12.0%,  14.1%] | 1.27* [1.22,1.33] | 18.4%  [17.1%,  19.9%] | 1.92* [1.83,2.01] | |
| Physician’s diagnosis & PHQ-9 cutoff score | Physician’s diagnosis only | 6.2% [6.1%,  6.3%] | 8.6% [8.5%,  8.7%] | 1.47*  [1.43,  1.50] | 9.5% [9.4%,  9.7%] | 1.76*  [1.73,  1.79] | |
|  | PHQ-9 ≥ 10 only | 6.0%  [5.9%,  6.1%] | 6.8%  [6.7%,  6.9%] | 1.21*  [1.20,  1.22] | 8.9% [8.7%,  9.0%] | 1.70*  [1.69,  1.71] | |
|  | Physician’s diagnosis & PHQ-9 ≥ 10 | 4.0% [3.9%,  4.1%] | 5.6% [5.5%,  5.7%] | 1.47*  [1.46, 1.48] | 8.7% [8.5%,  8.8%] | 2.48  (2.46, 2.50] | |

**S2 Frequencies, odds ratios and p-values for the associations between smoking status and lifetime & current depression**

*Note.* MINI = MINI International Neuropsychiatric Interview. PHQ-9 = Patient Health Questionnaire. OR = Odds ratio. p = p-value. Individuals who never smoked were used as reference group. All frequencies and odds ratios are reported with a confidence interval of 95%. **p* <10^−10^

**S3 Association of smoking status with lifetime depression by sex, age groups, and education level**

Associations were comparable between women (Physician’s diagnosis: OR_former_ = 1.46, 95% CI [1.40, 1.52]; OR_current_ = 1.92, 95% CI [1.84, 2.02]; MINI classification: OR_former_ = 1.42, 95% CI [1.31, 1.54]; OR_current_ = 1.89, 95% CI [1.72, 2.07], all *p* < 10^−10^) and men (Physician’s diagnosis: OR_former_ = 1.46, 95% CI [1.38, 1.54]; OR_current_ = 1.84, 95% CI [1.73, 1.95]; MINI classification: OR_former_ = 1.49, 95% CI [1.36, 1.63]; OR_current_ = 1.89, 95% CI [1.71, 2.09], all *p* < 10^−10^), with current smoker showing the strongest association with depression, followed by former (Figure S2-3, Tables S9A-B and S10A-B).

Across all age groups, current smokers (OR = 1.61-2.06, all *p <*10^−10^) showed the strongest association with depression, followed by former (OR = 1.35-1.86, all *p* < 10^−10^), compared to never-smokers for both physicians’ diagnosis and MINI classification (Figures S4-5). The strongest associations were observed for the age group 19-29 with relatively similar OR for former and current smokers (Physician’s diagnosis: OR_former_ = 1.86, 95% CI [1.61, 2.16]; OR_current_ = 2.06, 95% CI [1.81, 2.36]; MINI classification: OR_former_ = 1.86, 95% CI [1.46, 2.35]; OR_current_ = 1.95, 95% CI [1.58, 2.42]).  Meanwhile, the most pronounced differences between former and current smokers were observed in the age groups of 40-49 (Physician’s diagnosis: OR_former_ = 1.47, 95% CI [1.38, 1.57]; OR_current_ = 2.00, 95% CI [1.86, 2.14]; MINI classification: OR_former_ = 1.41, 95% CI [1.26, 1.58]; OR_current_ = 1.91, 95% CI [1.69, 2.16]) and 50-59 (Physician’s diagnosis: OR_former_ = 1.44, 95% CI [1.36, 1.53]; OR_current_ = 1.89, 95% CI [1.77, 2.02]; MINI classification: OR_former_ = 1.45, 95% CI [1.30, 1.62]; OR_current_ = 1.90, 95% CI [1.69, 2.15]). Detailed results can be found in Tables S9C-G and S10C-G.

Analyses stratified by education revealed similar results for low (Physician’s diagnosis: OR_former_ = 1.47, 95% CI [1.19, 1.81]; OR_current_ = 2.00, 95% CI [1.63, 2.46]; MINI classification: OR_former_ = 1.42, 95% CI [0.92, 2.20]; OR_current_ = 1.76, 95% CI [1.15, 2.71]), medium (Physician’s diagnosis: OR_former_ = 1.36, 95% CI [1.30, 1.43]; OR_current_ = 1.79, 95% CI [1.70, 1.89]; MINI classification: OR_former_ = 1.33, 95% CI [1.21, 1.46]; OR_current_ = 1.77, 95% CI [1.60, 1.95]) and high education (Physician’s diagnosis: OR_former_ = 1.50, 95% CI [1.43, 1.56]; OR_current_ = 1.92, 95% CI [1.82, 2.03]; MINI classification: OR_former_ = 1.49, 95% CI [1.37, 1.61]; OR_current_ = 1.94, 95% CI [1.76, 2.14]) for both physician’s diagnosis and MINI classification with current smokers showing the highest OR followed by former smokers compared to never smokers. For the education in progress, former smokers showed the highest OR followed by current smokers, compared to never smokers for both physician’s diagnosis (OR_former_ = 2.46, 95% CI [1.83, 3.27], OR_current_ = 2.23, 95% CI [1.72, 2.89]) and MINI classification (OR_former_ = 2.52, 95% CI [1.58, 3.95], OR_current_ = 2.32, 95% CI [1.53, 3.49]; Figures S6-S7, Tables S9H-K and S10H-K).

**S4 Association of smoking status with current depression by sex, age groups, and education level**

Associations between smoking status and current depressive symptoms were comparable between women and men, with current smoker showing the strongest association with depression (OR_female_ = 1.89, 95% CI [1.78, 2.01], OR_male_ = 1.94, 95% CI [1.80, 2.08], all *p <*10^−10^), followed by former (OR_female_ = 1.29, 95% CI [1.22, 1.36], OR_male_ = 1.25, 95% CI [1.16, 1.34], all *p <*10^−10^; Figure S8, Tables S11A-B).

Across all age groups, current smokers showed the highest OR for PHQ-9 cut-off ≥ 10 (OR = 1.39-2.24), followed by former smokers (OR = 1.08-1.48) compared to never smokers. The strongest asociations were observed in the age group 30-39 (OR_former_ = 1.48, 95% CI [1.15, 1.90]; OR_current_ = 2.24, 95% CI [1.73, 2.88]; Figure S9, Tables S11C-G).

Similar associations were observed across all education levels, with current smokers (OR = 1.69-2.29) showing the strongest associations, followed by former smokers (OR = 1.20-1.41). The strongest associations were observed for education in progress (OR_former_ = 1.41, 95% CI [1.05, 1.88]; OR_current_ = 2.29, 95% CI [1.82, 2.87]; Figure S10, Tables S11H-K).

**S5 Association of smoking intensity and severity of current depressive symptoms by sex, age groups, and education level**

Both sexes showed significant associations between cigarettes per day and PHQ-9 sum score, with comparable effect estimates for women (β = 0.07, 95% CI [0.06, 0.08], *p <*10^−10^) and men (β = 0.04, 95% CI [0.03, 0.04], *p <*10^−10^, Figure S11, Tables 12A-B). Across all age groups, cigarettes per day was significantly associated with PHQ-9 sum scores with consistent direction of effect estimates (β = 0.03-0.05, all *p <*10^−10^; Figure S12, Tables S12C-G). Similarly, cigarettes per day significantly predicted PHQ-9 sum scores across all education levels with a consistent direction of effect estimates (β = 0.03-0.06, all *p*< 0.004; Figure S13, Tables 12H-K).

**S6 Association of age at smoking initiation and depression onset by sex, age groups, and education level**

For both sexes, significant positive associations between the age at smoking initiation and depression onset were found, with larger effect estimates in women (β = 0.29, 95% CI [0.25, 0.33], *p <*10^−10^) than men (β = 0.18, 95% CI [0.13, 0.23], *p <*10^−10^, Figure S14, Tables S13A-B). For the age group-stratified analyses, significant positive associations between the age at smoking initiation and depression onset were observed across all age groups (β = 0.23-0.40, all *p <*10^−10^, Figure S15, Tables S13C-G) with the largest effect estimates for the age group 19-29 (β = 0.40, 95% CI [0.30, 0.50], *p <*10^−10^). For the education-stratified analyses, all of the associations were significant with comparable effect estimates (β = 0.24-0.33, all *p <*10^−10^) except for education in progress (*p* = 0.27, Figure S16, Tables S13H-K).

**S7 Association of time since smoking cessation and since the last depressive episode by sex, age groups, and education level**

Across both sexes, significant positive associations between time since smoking cessation and time since the last depressive episode were observed, with comparable effect estimates for women (β = 0.16, 95% CI [0.14, 0.18], *p <*10^−10^) and men (β = 0.18, 95% CI [0.16, 0.20], *p <*10^−10^, Figure S17, Tables S14A-B). For the age group-stratified analyses, significant positive associations were for all age groups with comparable effect estimates (β = 0.15-20, all *p <*10^−10^, Figure S18, Tables 14C-G). For the education-stratified analyses, significant positive associations were observed for all education levels (β = 0.10-0.19, *p <* 0.001, Figure S19, Tables 14H-K) except for education in progress (*p* = 0.19). The largest effect estimate was observed for high education (β = 0.19, 95% CI [0.17, 0.21], *p <*10^−10^).

**S8** **Association of time since smoking cessation and severity of current depressive symptoms by sex, age groups, and education level**

Across both sexes, significant negative associations between time since smoking cessation and PHQ-9 sum score were observed with equal effect estimates for women (β = -0.02, 95% CI [-0.02, -0.02], *p < 10^−10^*) and men (β = -0.02, 95% CI [-0.03, -0.02], *p < 10^−10^*, Figure S20, Tables 15A-B). For the age group-stratified analyses, except for the age group 19-29, significant negative associations were observed for the other age groups with small effect estimates (β = -0.02-0.03, all *p <*0.001, Figure S21, Tables S15C-G). For the education-stratified analyses, except for education in progress (p = 0.07), significant negative associations with small effect estimates were observed for the other education levels (β = -0.02-0.04, all *p <*004, Figure S22, Tables S15H-K).
